# Autophagic flux inhibition and lysosomogenesis ensuing cellular capture and retention of the cationic drug quinacrine in murine models

**DOI:** 10.7717/peerj.1314

**Published:** 2015-10-06

**Authors:** Alexandre Parks, Xavier Charest-Morin, Michael Boivin-Welch, Johanne Bouthillier, Francois Marceau

**Affiliations:** Research Center CHU de Québec, Université Laval, Quebec City QC, Canada

**Keywords:** Quinacrine, V-ATPase, Drug reservoirs, Lysosomogenesis, TFEB, Macroautophagy, Ion trapping

## Abstract

The proton pump vacuolar (V)-ATPase is the driving force that mediates the concentration of cationic drugs (weak bases) in the late endosome-lysosome continuum; secondary cell reactions include the protracted transformation of enlarged vacuoles into autophagosomes. We used the inherently fluorescent tertiary amine quinacrine in murine models to further assess the accumulation and signaling associated with cation trapping. Primary fibroblasts concentrate quinacrine ∼5,000-fold from their culture medium (*K_M_* 9.8 µM; transport studies). The drug is present in perinuclear granules that are mostly positive for Rab7 and LAMP1 (microscopy). Both drug uptake and retention are extensively inhibited by treatments with the V-ATPase inhibitor bafilomycin A1. The H^+^ ionophore monensin also prevented quinacrine concentration by fibroblasts. However, inhibition of plasma membrane transporters or of the autophagic process with spautin-1 did not alter quinacrine transport parameters. Ancillary experiments did not support that low micromolar concentrations of quinacrine are substrates for organic cation transporters-1 to -3 or P-glycoprotein. The secondary autophagy induced by quinacrine in cells may derive from the accumulation of incompetent autophagolysosomes, as judged from the accumulation of p62/SQSTM1 and LC3 II (immunoblots). Accordingly, protracted lysosomogenesis is evidenced by increased expression of LAMP1 and LAMP2 in quinacrine-treated fibroblasts (48 h, immunoblots), a response that follows the nuclear translocation of the lysosomal genesis transcription factor TFEB and upregulation of LAMP1 and −2 mRNAs (24 h). Quinacrine administration to live mice evidenced variable distribution to various organs and heterogeneous accumulation within the lung (stereo-microscopy, extraction). Dose-dependent *in vivo* autophagic and lysosomal accumulation was observed in the lung (immunoblots). No evidence has been found for transport or extrusion mechanisms modulating the cellular uptake of micromolar quinacrine at the plasma membrane level. As shown *in vitro* and *in vivo*, V-ATPase-mediated cation sequestration is associated, above a certain threshold, to autophagic flux inhibition and feed-back lysosomogenesis.

## Introduction

The concentration of cationic drugs (weak bases) in acidic cell compartments is well documented, at least in cultured cells (De Duve et al., 1974; Marceau et al., 2012) and freshly isolated peripheral blood leukocytes (Roy et al., 2013). A general model is emerging: the proton pump vacuolar (V)-ATPase is the driving force that mediates the concentration and retention of such amines, mainly in the late endosome-lysosome continuum. Indeed, potent and specific V-ATPase inhibitors, such as bafilomycin A1, prevent cellular drug accumulation and initiate drug release in preloaded cells. The ion trapping theory holds that amine drugs become protonated and incapable of retro-diffusion in organelles that are constantly acidified; secondary cell reactions include the osmotic expansion of vacuoles and their protracted (1–4 h) transformation into autophagosomes (Marceau et al., 2012).

While the ion trapping model of the cellular concentration of organic amines has been verified in a number of cell types, many issues in this model remain uncertain or not widely verified:

(1) It is not known whether the autophagic process originates from organelle damage, from cell starvation (apparently not the case as monitored by phospho-AKT levels in cells treated with local anesthetics, Bawolak, Morissette & Marceau, 2010), or from an inhibition of the “autophagic flux” with the accumulation of incompetent autophagolysomomes. There is limited evidence for the latter process in cells treated with desmethylclomipramine or chloroquine, based on the accumulation of p62/SQSTM1, a protein cleared by autophagy (Iwai-Kanai et al., 2008; Rossi et al., 2009; Sheen et al., 2011), or based on the vacuolar accumulation of tagged recombinant LC3 in cells treated with norephedrine (Funakoshi et al., 2013).
(2) The influence of the presumably secondary autophagic process on the upstream cationic drug uptake has not been studied.
(3) If the concentration of cationic drugs inactivates in part the lysosomal function via pH buffering (Funakoshi et al., 2013), a possible feed-back lysosomogenesis may ensue. This mechanism is suggested by the upregulation of the lysosomal glycoproteins LAMP1 and LAMP2 in cells treated with some cationic drugs, imatinib or chloroquine (Ertmer et al., 2007; Chen, Gombart & Chen, 2011). Ultimately, the process may be controlled by the lysosomal genesis transcription factor TFEB that is subjected to nuclear translocation upon activation (Sardiello et al., 2009) and such a protracted response to basic drugs has been recently reported in cultured cells (chloroquine: Roczniak-Fergusson et al., 2012; propranolol: Logan, Kong & Krise, 2014).
(4) Intriguing tissue or cell type specificities were described for the formation of cationic drug reservoirs. Thus, *in vitro*, macrophages concentrate more amiodarone than do smooth muscle cells (Morissette et al., 2009); freshly isolated neutrophils exhibit a higher affinity but lower *V*_max_ than lymphocytes for the transport of quinacrine (Roy et al., 2013). *In vivo*, the lipophilic amine clofazimine is redistributed from adipose tissue to macrophage-rich organs, such as the spleen and liver, as a function of time (Baik et al., 2013). Limited evidence also supports that quinacrine binds to or is transported by organic cation transporter (OCT)-2 (Sweet & Pritchard, 1999) and P-glycoprotein (PGP) (Dohgu et al., 2004; Beck et al., 1988; Huang et al., 2006), a possible basis for differential tissue distribution via facilitation of drug uptake or extrusion.

In previous studies, we have exploited the inherently fluorescent drug quinacrine to perform quantitative transport and morphological studies of human cells (Marceau et al., 2009; Roy et al., 2013; Marceau, Roy & Bouthillier, 2014). Formerly used as an antiprotozoal drug, quinacrine considerably accumulates over time during chronic dosing in tissues such as the liver, lungs and spleen *in vivo* (Huang et al., 2006; Ehsanian, Van Waes & Feller, 2011). Tissue reservoirs of this drug are only slowly released upon cessation of dosing. Quinacrine has been exploited in murine models with several objectives:

(1) To document tissue and subcellular distribution of the drug, as assessed by its intrinsic fluorescence in a murine model, and possible tissue specific modulation of reservoir formation by plasma membrane transporters.
(2) To further characterize the autophagic accumulation in cells that concentrate quinacrine.
(3) To evidence the possible lysosomogenesis in response to the buffering of acidic organelles by quinacrine concentrated from the extracellular fluid.
(4) To confirm *in vivo* the general model derived from *in vitro* experiments (cellular quinacrine uptake dependent on V-ATPase, leading to autophagic accumulation and lysosomogenesis).

## Materials and Methods

### Animals and cells

A local ethics committee approved the use of C57BL/6 male mice (Charles River, St. Constant, QC, Canada), 8 weeks old, for the isolation of cells and tissues and *in vivo* quinacrine administration (CHU de Québec Animal Protection Committee, approval numbers 12-073 and 2015-029). Following euthanasia with a combination of isoflurane and CO_2_, dermal fibroblasts were cultured from multiple explants in Dulbecco’s Modified Eagle Medium (Life Technologies) supplemented with 10% fetal bovine serum (FBS) and antibiotics and propagated, up to passage 6, in the same medium. Several primary fibroblast lines were obtained from various mice. Macrophages from broncho-alveolar lavage were isolated from mice as a suspension in PBS containing 0.2% EDTA (Boilard et al., 2014), centrifuged (1,500 rpm, 10 min), resuspended in EMEM supplemented with 10% FBS at a density of 33,000 cells/ml, and further purified by adherence to 35 mm petri dishes (2 ml cell suspension/petri; 1 h incubation at 37 °C, 5% CO_2_). Cell dishes were further treated with drugs for 4 h (37 °C), rinsed with PBS and observed (fluorescence and transmission microscopy).

*In vivo* treatment with quinacrine was applied to some mice under the form of 2 intraperitoneal injections (40 or 80 mg/kg twice, 24 h apart, followed by sacrifice 24 h after the last injection). Control mice received the drug vehicle (100 µl warm saline). These dose levels are documented and tolerated in mice (Gorbachev et al., 2007). These mice were used (1) for the detection of quinacrine fluorescence in freshly dissected organs (fluorescence stereo-microscope, Leica, model MZ APO; filter set: excitation 480 nm/40 nm, emission 510 nm long pass; coupled to the Olympus DP73 camera), (2) to quantify quinacrine fluorescence in the NaOH, 1 N, extracts of mouse organ as outlined below, and (3) to gather lung tissue, homogenized for immunoblotting of p62/SQSTM1 and LAMP2 (performed as described below for cell extracts). To this end, the mouse organs were homogenized using a glass–glass pestle in a buffer containing 150 mM NaCl, 1% Triton X-100, 0.1% SDS, 0.5% Na deoxycholate, 50 mM Tris and one Complete Mini Protease inhibitor cocktail tablet (Roche) per 10 ml. 100 µg of protein were loaded in each SDS-PAGE track.

### Drugs

Bafilomycin A1 was purchased from LC Laboratories (Woburn, MA). Other drugs, from Sigma-Aldrich (St. Louis, MO), included the fluorescent agents quinacrine dihydrochloride and doxorubicin hydrochloride. Other significant drugs from the same source were the macroautophagy inhibitor spautin-1 (Liu et al., 2011; Shao et al., 2014), the H^+^ ionophore monensin (Gil et al., 2012), the inhibitor of OCT-1 to -3, decynium-22 (Hayer-Zillgen, Brüss & Bönisch, 2002), elacridar (GF120918, a high potency P glycoprotein inhibitor; Rautio et al., 2006) and promiscuous transporter inhibitors (gemfibrozil, verapamil, *β*-estradiol, cetirizine).

### Generation of the LAMP1-mCherry vector

First, the sequence corresponding to the human LAMP1 protein was amplified from the previously described LAMP1-GFP vector (gift from Dr. Esteban C. Dell’Angelica, UCLA, Department of Human Genetics; Falcón-Pérez et al., 2005) using the following PCR primers: 5′-C GTT TAA ACG GGC CCT ATG GCG GCC CCC GGC AGC-3′ (sense) and 5′-TTG CTC ACC GCG CCG GTG GAG CCT GTG-3′ (antisense). Then, the sequence coding for the mCherry was in turn amplified with the following primers: 5′-CCG GCG CGG TGA GCA AGG GCG AGG AG-3′ (sense) and 5′-TTG GTA CCG AGC TCG TTA CTT GTA CAG CTC GTC CAT GC-3′ (antisense). Following purification, both fragments were ligated in the XbaI/BamHI digestion product of the pcDNA3.1(-) vector using the Gibson assembly technique to generate the LAMP1-mCherry vector. This vector was validated by sequencing.

### Vectors and cell transfection

Mouse dermal fibroblasts were transiently transfected with several vectors to study the subcellular localization of quinacrine (itself detected using its intrinsic green fluorescence) or the signaling induced by quinacrine treatment. Thus, vectors encoding Rab5, Rab7 and Rab7-GTP-locked fused to the mCherry fluorescent protein were gifts from Drs. Robert Lodge (Institut de recherches cliniques de Montréal, Montreal, Canada) and Michel J. Tremblay (CHU de Québec, Quebec City, Canada). Endoplasmic reticulum-targeted red fluorescent protein (ER-RFP; Klee & Pimentel-Muiños, 2005) was a generous gift from Dr. Felipe X. Pimentel-Muiños, Universidad de Salamanca-CSIS, Salamanca, Spain. A vector encoding the myc-tagged form of the transcription factor TFEB was purchased from Origene (cat. no. RC207153). Expression vectors were transiently transfected for 24 or 48 h using the polyethylenimine (PEI) transfection reagent protocol (Morissette et al., 2008) or, for TFEB, using the X-tremeGENE reagent (Roche), used as directed.

### Microscopy

In live cultured murine fibroblasts, the subcellular distribution of quinacrine was monitored after various treatments at 37 °C (30 min-4 h) using its intrinsic green epifluorescence. The colocalization of red fluorescent proteins was monitored in some experiments. Mitochondrial staining was applied as 25 nM MitoTracker Red CMXRos (Invitrogen) added to the culture medium 15 min before microscopic observation. Immunofluorescence in fixed and permeabilized cells was applied for detecting *α*-actin (monoclonal antibodies, clone 1A4, Sigma-Aldrich, dilution 1:100; revealed with anti-mouse IgG polyclonal antibodies coupled to AlexaFluor-488. Molecular Probes) and the subcellular distribution of myc-tagged TFEB (anti-myc monoclonal antibodies, clone 4A6, conjugated with AlexaFluor-488, Millipore). Cells were photographed using an Olympus BX51 microscope coupled to a CoolSnap HQ digital camera (filters for quinacrine and AlexaFluor-488: excitation 460–500 nm, emission 510–560 nm; for mCherry, RFP and Mitotracker Red: excitation 525–555 nm, emission 600–660).

### Cell transport

The uptake of quinacrine in mouse cells was established using a slight variation of a technique previously applied to a different cell type (Marceau et al., 2009). Briefly, test drugs were added to confluent 25 cm^2^ cell flasks containing 3 ml of serum-containing culture medium according to various schemes and time frames. Quinacrine uptake at the end of the allowed incubation period (37 °C, 5% CO_2_) was determined by rapidly washing each cell flask 3 times with 3 ml of phosphate-buffered saline, pH 7.4, at room temperature and dissolving the cells with 10 ml of 1 N NaOH. Quinacrine content was analyzed in the NaOH extract using a fluorescence spectrophotometer (either FluoroLog tau-3; HORIBA JobinYvon Inc., Edison, NJ or Aminco Bowman Series 2) against a standard curve of the authentic drug dissolved in NaOH 1 N (excitation 414 nm, emission 501 nm). Control fluorescence from extracts of untreated cells was systematically verified, of small magnitude and subtracted from experimental values.

The same fluorometric assay was applied to selected organs of mice dosed *in vivo* with quinacrine (40 mg/kg/day i.p. for 2 days) or its saline vehicle. To this end, the mouse organs were rapidly homogenized using a glass–glass pestle in 1 N NaOH (10 ml per organ, further diluted with NaOH as needed, fluorescence measured as outlined above). The autofluorescence cannot be subtracted in this experimental setting, but may be quantified from the saline-treated animals.

### Immunoblots

For the analysis based on cultured fibroblasts, 25-cm^2^ flasks of confluent cells were treated for 24–48 h with quinacrine, other drugs or serum starvation to monitor effects on proteins affected by autophagy (LC3, p62/SQSTM1) or lysosomogenesis (LAMP1, LAMP2). The cells were then put in boiling lysis buffer containing 10 mmol/L Tris, pH 7.4, 1.0 mmol/L Na_3_VO_4_, and 1.0% SDS. The lysates were centrifuged at 15,000 g for 5 min and incubated for 5 min at 95 °C. Total protein concentrations were then determined with the use of the BCA Protein Assay (Pierce). Fifteen micrograms of total protein was run on a SDS-PAGE and transferred to a PVDF membrane. Some extracts for immunoblots were prepared from homogenized mouse organs, as outlined above, and similarly processed. Anti-human LC3B rabbit polyclonal antibodies (Novus; dilution 1:3000) were used to reveal the cytosolic form LC3 I (18 kDa) and the lipidated and membrane-bound form LC3 II in total cell extracts run on 15% SDS-PAGE and transferred to PVDF membranes (Morissette, Lodge & Marceau, 2008). Nine % SDS-PAGE was used to separate constituents from samples subsequently revealed using antibodies specific for p62/SQSTM1, LAMP1, LAMP2 and *β*-actin. p62/SQSTM1 rabbit monoclonal antibodies were from Cell Signaling Technology (cat. no. 5114). The lysosomal/late endosomal glycoproteins LAMP1 and LAMP2 were detected in total cell extracts using the rat monoclonal antibodies 1D4B and ABL-93, respectively (dilution 1:1,000 for each, Developmental Studies Hybridoma Bank, Iowa City, IA) revealed using an HRP-conjugated anti rat IgG. The primary antibodies are specific to the mouse LAMP proteins. All reactions involved HRP-labelled secondary antibodies revealed using a luminescent substrate used as directed (Western Lightning, PerkinElmer) with CL-X Posure film (Thermo Scientific). Equal track loading was verified by separating and transferring the same samples separately and immunoblotting for *β*-actin (mouse monoclonal from Sigma-Aldrich, dilution 1:50,000). Intensities of immunoblot signals were derived from scanned versions of photographic films: the mean pixel intensity (0–255 scale) of rectangular selections corresponding to migrated proteins was evaluated using the Photoshop software (version 6, Adobe, San Jose, CA). The background signal was subtracted.

### RNA isolation

Mouse dermal fibroblasts were plated in 6-well plates two days prior to stimulation. Total RNA was extracted using Qiazol (Qiagen Inc., Mississauga, ON, Canada) according to the manufacturer’s protocol, with modifications. Briefly, after 24 h of treatment, cells were washed twice with room temperature PBS before adding 1 ml of Qiazol in each well. Cells were detached using a cell scrapper and the suspension was transferred to a tube containing 200 µL of chloroform. Afterwards, samples were vigorously mixed and then centrifuged at 12,000 g for 15 min (4 °C). The aqueous phase (≈400 µL) was transferred to a 1.5 ml Eppendorf tube containing an equal volume of isopropanol. After 10 min of incubation at room temperature, the samples were centrifuged for 10 min at 12,000 g (4 °C). The supernatant was discarded and the RNA pellet was washed twice using 500 µL of 70% ethanol followed by a 5 min centrifugation (12,000 g, 4 °C). The final pellet was air-dried for 10–15 min and then re-suspended in RNAse-free water. The resulting RNA solution was then quantified using a Biodrop µLITE instrument.

### Comparative real-time PCR

The mRNA transcript level was monitored in mouse dermal fibroblasts using real-time PCR as described by Laflamme et al. (2014). Briefly, reverse transcription was performed using 1 µg of total RNA with a Transcriptor First Strand cDNA Synthesis Kit (Roche Applied Science, Laval, QC, Canada) following the manufacturer’s instructions. Complementary DNA amplifications were performed in a Rotor-Gene Q operated with Q-series software version 2.0.2 (Qiagen Inc., Mississauga, ON, Canada) using 35 cycles of 95 °C for 17 s, 58 °C for 25 s and 72 °C for 25 s. Each reaction mixture contained 40 ng of cDNA, 2 µL of 10× Buffer, 100 µM of dNTPs, 0.1 unit of Taq DNA polymerase (Roche Applied Science) and SYBR Green I (Life Technologies Inc., Burlington, ON, Canada) diluted 1:30,000. For each murine gene of interest, specific primers were designed following certain specifications as described by Laflamme et al. (2014). For the LAMP1 gene, the following primers were used: 5′-ACTGGTAACAACGGAACCTG-3′ (forward) and 5′-ACACATTGGGGTTAGGAACA-3′ (reverse). For the LAMP2 gene, we used the following primers: 5′-CTAGGAGCCGTTCAGTCCAA-3′ (forward) and 5′-CTTGCAGGTGAATACCCCAA-3′ (reverse). GAPDH was used as a reference gene for the normalization of results using the following primers: 5′-AACTTTGGCATTGTAGAAGG-3′ (forward) and 5′-ACACATTGGGGTTAGGAACA-3′ (reverse).

### Data analysis

Numerical results are presented as mean ± S.E.M. Quinacrine transport data obtained by varying the drug concentration during a fixed incubation period were fitted by non-linear regression to the Michaelis–Menten equation using a least-square method (Prism4.0; GraphPadSoftware Inc., San Diego, CA, USA) and *K*_M_ and *V*_max_ values calculated from this procedure. Sets of numerical data obtained via immunoblot densitometry, cytofluorometric intensities, enzyme activity, or MPP^+^ transport were generally compared by ANOVA followed Bonferroni multiple comparison tests to compare selected pairs of values, of by Dunnett’s test to compare experimental groups with a common control value. If a set of values was normalized (control = 1), the non-parametric Kruskal–Wallis ANOVA and Dunn’s multiple comparison test were applied instead. The qualitative effect of treatments on the subcellular distribution of TFEB (either predominantly cytosolic or nuclear) was evaluated for each stained fibroblast in microphotographic records and proportions of affected cells were compared using the *χ*^2^ statistics to determine drug effect. Fluorescence intensity in organ extracts from mice treated with either saline or quinacrine was compared using *t* test. All computations were performed using the InStat 3.05 computer program, GraphPad Software (San Diego, CA).

## Results

### Quinacrine transport by murine fibroblasts

**Figure 1:**
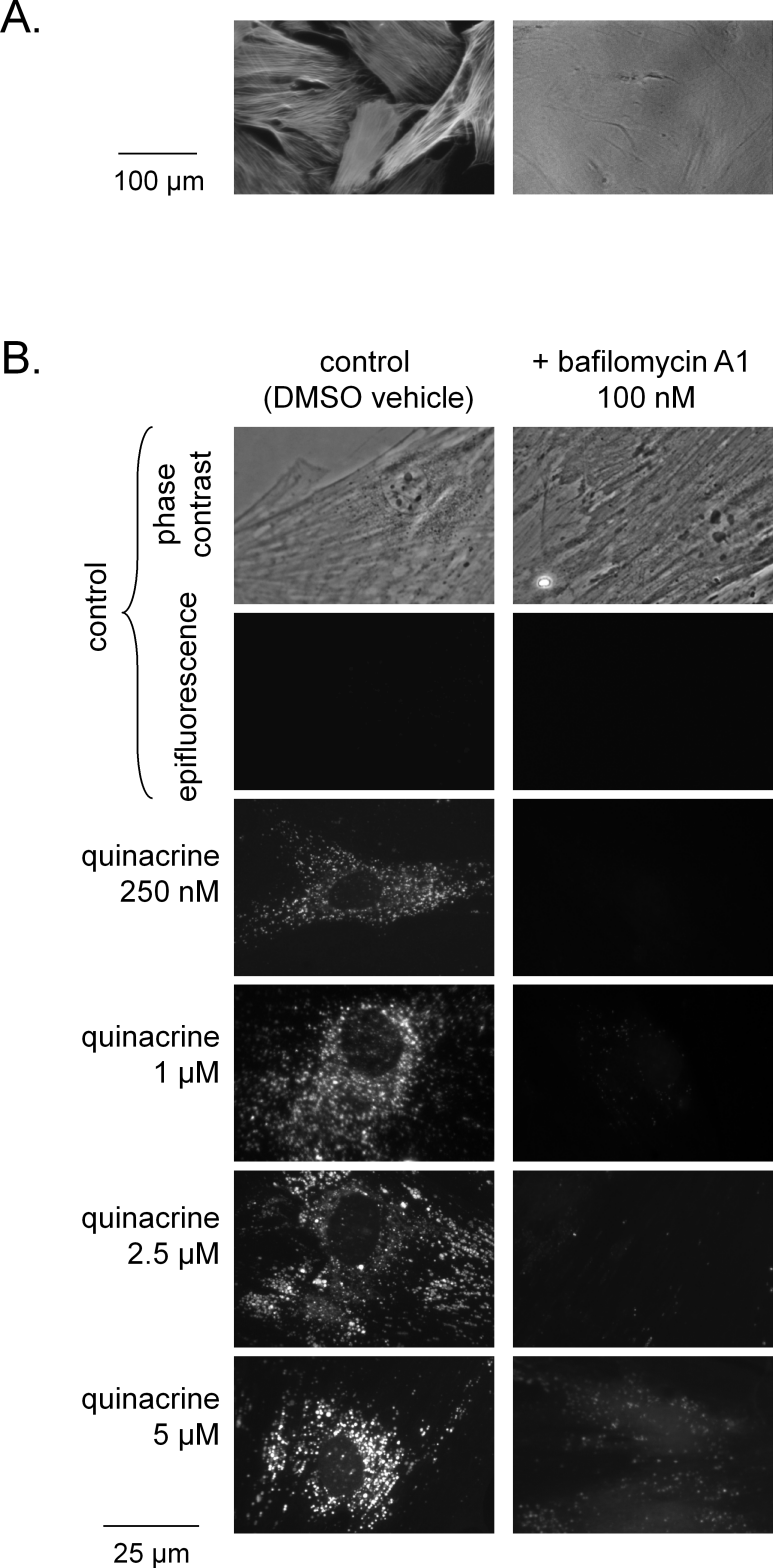
Microscopic evidence of quinacrine accumulation C57BL/6 mouse dermal fibroblasts. (A) Uniform expression of smooth muscle *α*-actin in these cells (immunofluorescence, left; right: same field in phase contrast; 100×). (B) Granular uptake of quinacrine (250 nM–5 µM, 4 h) into dermal fibroblasts and effect of co-treatment with bafilomycin A1 (100 nM) (green epifluorescence; a matched phase contrast view is shown for the control field). Original magnification 400×.

The primary dermal fibroblasts from C57BL/6 mice were uniformly positive for smooth muscle *α*-actin distributed as stress fibers (immunofluorescence, Fig. 1A), thus qualifying more precisely as myofibroblasts (Hinz, 2007). As reported for various cells of human origin (Marceau et al., 2009; Marceau, Roy & Bouthillier, 2014; Roy et al., 2013), these cells from wild type C57BL/6 mice accumulated quinacrine under the form of perinuclear and cytosolic granules and vacuoles, depending on the drug concentration (250 nM–5 µM), as judged by the intrinsic green fluorescence of the drug (Fig. 1B). The nuclear staining was negligible in the quinacrine concentration range tested. As with the human cells again, co-treatment of murine cells with the V-ATPase specific inhibitor bafilomycin A1 virtually abolished drug uptake (Fig. 1B).

**Figure 2:**
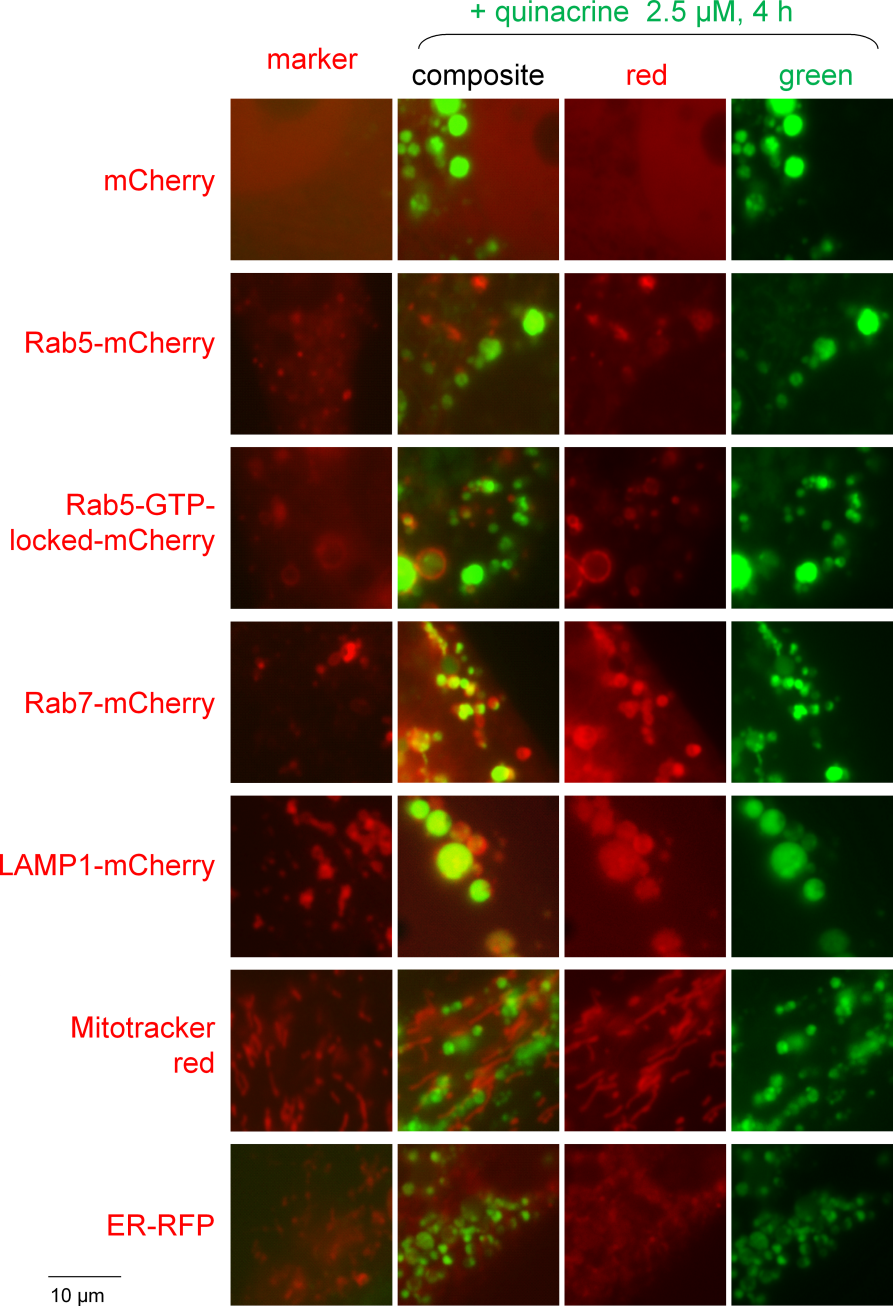
Colocalization studies of red fluorescent markers with quinacrine in mouse fibroblasts treated as indicated. All the red markers are fusion proteins with a red fluorescent protein except for Mitotracker red. Original magnification 1,000×. Colocalization is strong for Rab7 and LAMP1, occasional for Rab5 and negative for the other markers.

The identity of the organelles that concentrated quinacrine in murine fibroblasts was investigated in cells transiently expressing a marker protein fused to a red fluorescent protein or treated with Mitotracker red (Fig. 2). While the green fluorescence of quinacrine was highly colocalized with the Rab7 and LAMP1 fusion proteins, constituents of late endosomes and lysosomes, it was occasionally seen along Rab5, an early endosome marker. However, an activated (GTP-locked) Rab5 construction, well known to cause the formation of giant hollow vacuoles where endocytosis cargo accumulate (Stenmark et al., 1994), was also often colocalized with quinacrine in a particular way, the drug being inside the giant vacuoles while the red protein lined them (Fig. 2). These results support a certain diversity of the organelles that concentrate cationic drugs by the mediation of V-ATPase. Mitotracker red and an endoplasmic reticulum-targeted red fluorescent protein were not colocalized with quinacrine-rich organelles.

**Figure 3:**
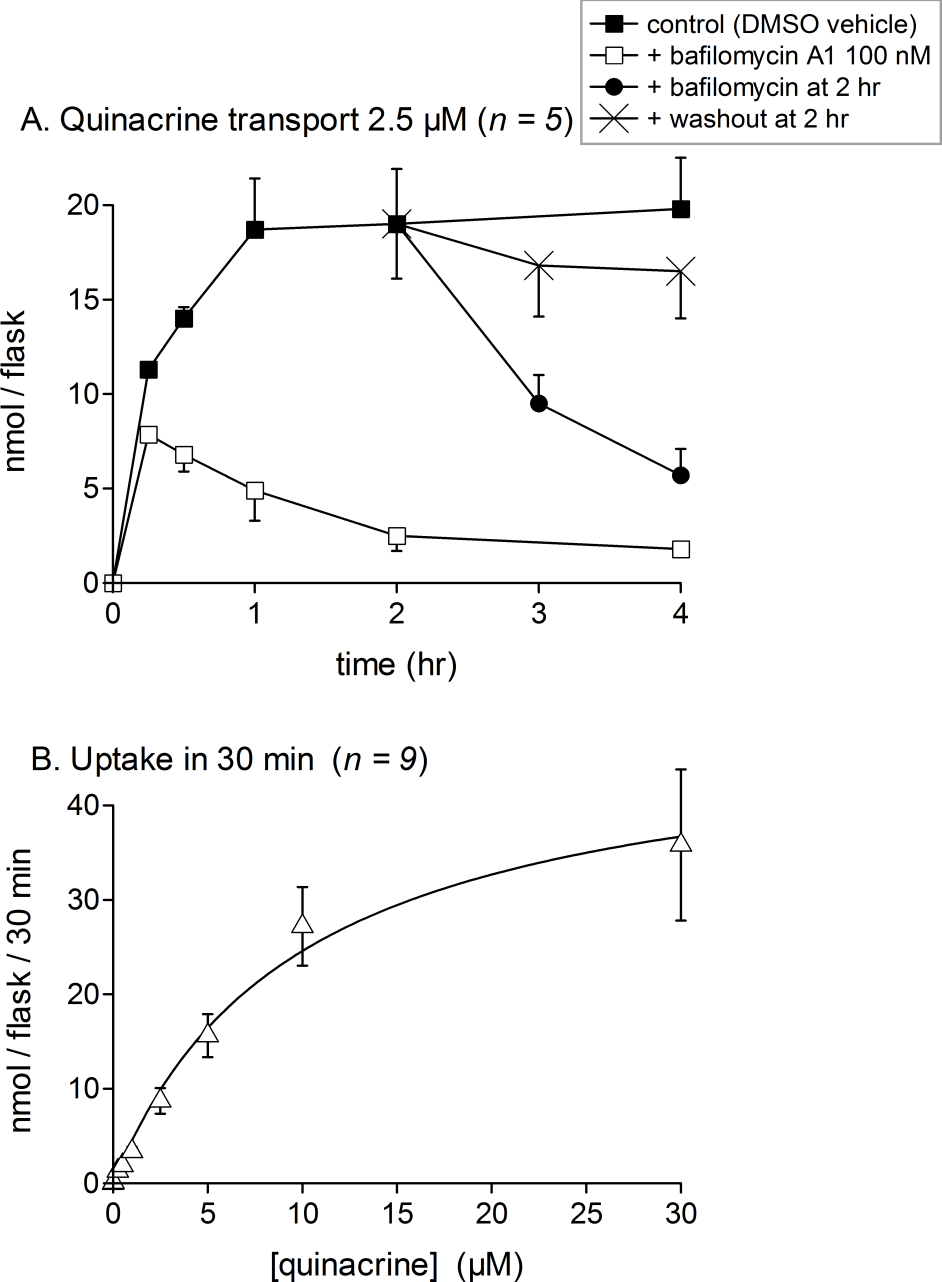
Uptake of quinacrine into confluent C57BL/6 mouse dermal fibroblasts as measured using extract-associated fluorescence. Each experimental point is derived from a confluent 25-cm^2^ flask. Values are mean ± s.e.m. of the number of replicates indicated by *n*. (A) Time course of drug uptake and drug retention upon bafilomycin A1 (100 nM) addition or drug washout for a 2.5 µM drug concentration. (B) Effect of drug concentration on uptake during a 30-min period (described in Results).

**Figure 4:**
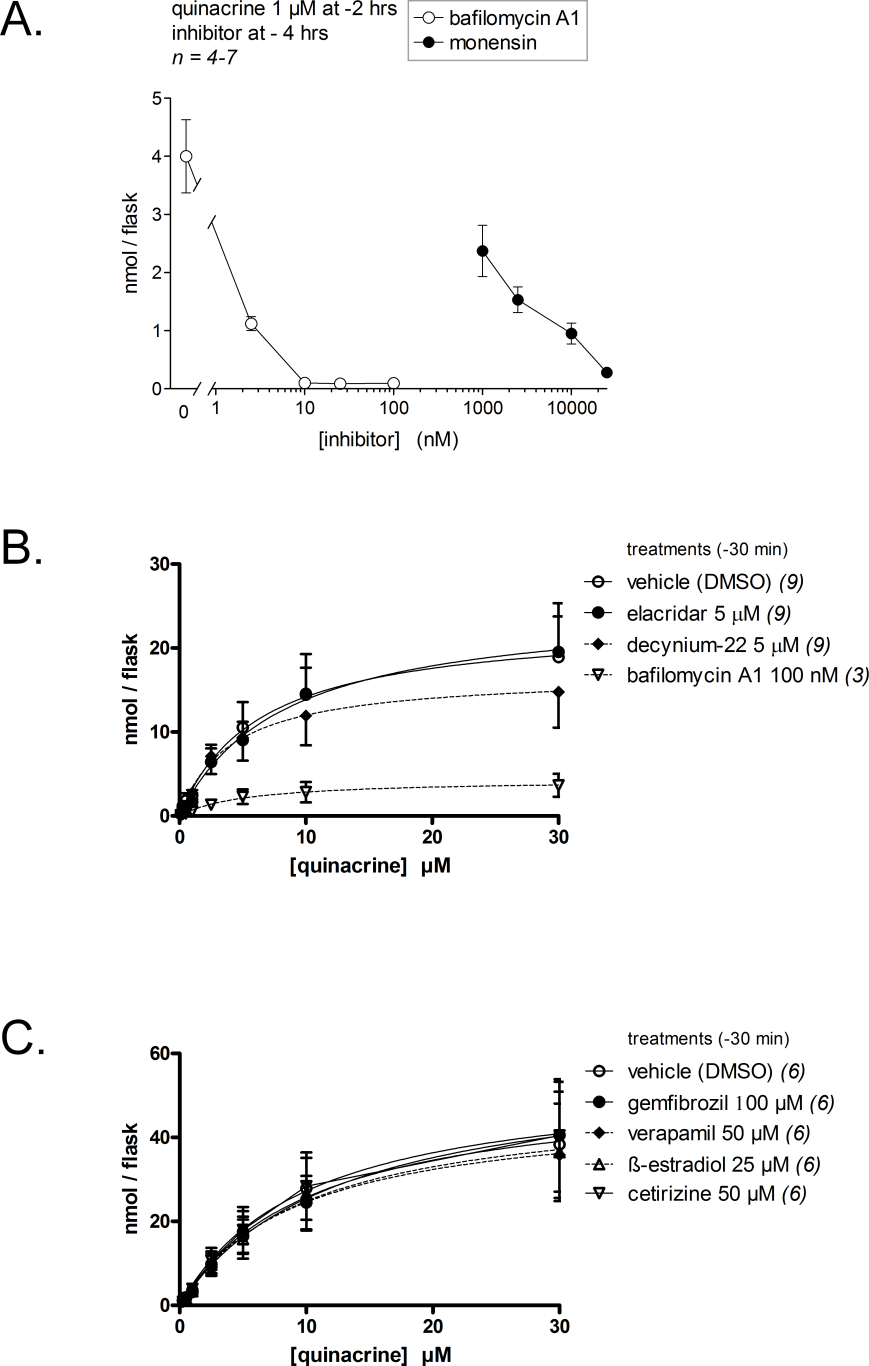
Effect of inhibitory drugs on quinacrine accumulation by dermal fibroblasts. (A) Concentration-effect relationship for bafilomycin- and monensin-induced suppression of quinacrine uptake (1 µM, 2 h) by mouse dermal fibroblasts. Presentation as in Fig. 3A. Experimental values were compared using ANOVA (*P* < 10^−4^) followed by Dunnett’s test to compare each value from inhibitor-treated cells with the control uptake value without inhibitor (the effect of all concentrations of either inhibitor was significant, *P* < 0.01). (B) Effect of the P-glycoprotein inhibitor elacridar, of the OCT-1 to −3 inhibitor decynium-22 and of bafilomycin A1 on acute (30 min) quinacrine accumulation by fibroblasts (inhibitory drugs were applied 30 min before quinacrine). Values are means ± SEM of the number of determinations indicated between parentheses. (C) Effect of “promiscuous” transport inhibitors (concentrations as indicated) on quinacrine uptake by fibroblasts (same protocol as in B). Presentation as in (B).

Quantifying quinacrine fluorescence in cell extracts allowed a more detailed characterization of the transport of this drug for up to 4 h (Fig. 3). Quinacrine, at the low 2.5-µM concentration in the culture medium, was taken up efficiently by cells, reaching saturation in 60 min. Cell co-treatment with bafilomycin A1 inhibited the uptake of quinacrine for all tested incubation periods, but most importantly at later times, as if the inhibitor had a protracted effect on the pH of acidic organelles (Fig. 3A). In cells loaded for 2 h with quinacrine, most of the cell-associated drug was released after the addition of bafilomycin A1, but not after drug washout with fresh culture medium, in which case the retention of the drug was extensive. The uptake of quinacrine for a short 30-min period followed an apparent hyperbolic kinetics with an apparent *K*_m_ of 9.8 µM (8.7 µM in human smooth muscle cells, Marceau et al., 2009) and a *V*_max_ of 48.7 nmol/flask/30 min (Fig. 3B; 29 nmol/flask in human smooth muscle cells). One flask of mouse fibroblasts contains approximately 276,000 ± 43,000 cells (*n* = 6), corresponding to a packed cell volume of 5–7 µl, indicating a ∼5,000-fold concentration of quinacrine in the cell compartment, relative to the drug concentration in the culture medium. The concentration-effect relationship of bafilomycin A1-induced suppression of quinacrine uptake by fibroblasts has been explored for a fixed quinacrine and low concentration and a 2-hr treatment duration (1 µM; Fig. 4A). A low-nanomolar potency was observed for bafilomycin A1. Consistent with the ion trapping model, the H^+^ ionophore monensin, that disrupts the organelle proton gradients at micromolar concentration levels (Gil et al., 2012), also profoundly prevented the cell uptake of quinacrine (Fig. 4A). In these experiments where only 1 µM of quinacrine was used, the residual quinacrine measured in the presence of 100 nM of bafilomycin A1 was lower than that measured in cells treated with a higher concentration of quinacrine (compare Figs. 3A and 4A). In short-term experiments involving inhibitors of vacuolar pH gradients (Figs. 1, 3A and 4A), no cytotoxicity was apparent. When applied 30 min before quinacrine, bafilomycin A1 extensively inhibited cellular drug uptake over all the quinacrine concentration range (Fig. 4B).

As seen in other cellular models, the inhibitor of OCT-1 to −3, decynium-22 (Hayer-Zillgen, Brüss & Bönisch, 2002), failed to significantly modify the acute (30 min) transport of quinacrine into murine fibroblasts (Fig. 4B). Elacridar (GF120918), a high potency P glycoprotein inhibitor (Rautio et al., 2006), also failed to significantly modify quinacrine uptake in murine fibroblasts (Fig. 4B), ruling out another type of modulation of this process by transporter molecules located at the plasma membrane. These findings were corroborated in additional quinacrine transport studies that involved “promiscuous” transporter inhibitors used at high concentrations (≥25 µM). Thus, gemfibrozil, verapamil, *β*-estradiol or cetirizine pre- and co-treatment did not modify quinacrine uptake by mouse fibroblasts (Fig. 4C). At the chosen concentration levels, gemfibrozil inhibits at least PGP, organic anion-transporting polypeptide (OATP) 1B1 and OATP1B3 (Seral et al., 2003; Jeong et al., 2015); the wide spectrum of verapamil covers PGP, OCT-1, OCTN-1 and OCTN-2 (Salomon et al., 2014); *β*-estradiol inhibits OCT-3 and OCT-1 (Hayer-Zillgen, Brüss & Bönisch, 2002); cetirizine inhibits at least PGP, OCT-2 and OCTN-2 (Tsuruoka et al., 2006; Diao, Ekins & Polli, 2010).

### Autophagic accumulation in quinacrine-treated murine fibroblasts

**Figure 5:**
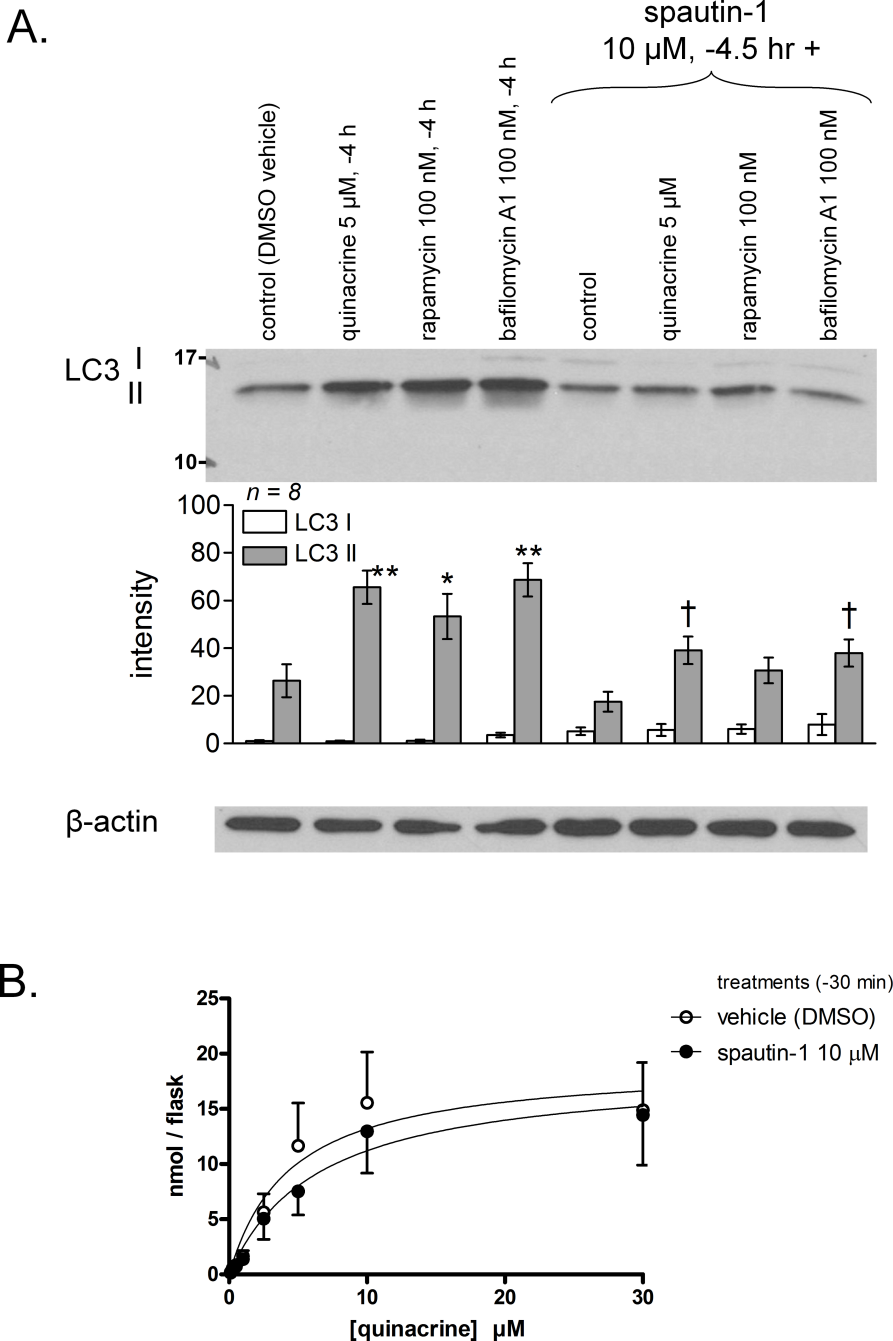
Effect of the autophagy inhibitor spautin-1 on quinacrine transport in mouse fibroblasts. (A) Validation of spautin-1 as an inhibitor of LC3 II accumulation in cells treated for 4 h with drugs. Histograms represent average densitometry values of immunoblots for replicated experiments. LC3 I cell concentration did not significantly differ (ANOVA). LC3 II values were heterogeneous (*P* < 0.001; Bonferroni multiple comparison tests for selected pairs: ^∗^ *P* < 0.05, ^∗∗^ *P* < 0.001 vs. control; ^†^ *P* < 0.05 vs. same treatment without spautin-1). (B) Effect of spautin-1 on quinacrine accumulation measured over a duration (4 h) that supports quinacrine-induced autophagy (replicate number = 5). In (A) and (B), spautin-1 (10 µM) was applied 30 min before other drugs.

Macroautophagy is activated in cells treated with the mTor inhibitor rapamycin, that mimics metabolic deprivation (Codogno & Meiler, 2005), but autophagic flux inhibition is the suspected mechanism by which amines or bafilomycin A1 lead to autophagosome accumulation (Marceau et al., 2012). This was replicated in primary murine fibroblasts, as assessed by the accumulation of the activated and lipidated form (II) of the macroautophagic effector LC3 in response to 5 µM quinacrine and the other drugs (see ‘Introduction’) (Fig. 5A). Spautin-1 is an effective inhibitor of autophagy that works by downregulating beclin-1 upstream of membrane envelopment of autophagosomes (Liu et al., 2011; Shao et al., 2014). The drug significantly inhibited the cellular LC3 II accumulation induced in 4 h by quinacrine or bafilomycin A1 treatments while somewhat increasing the concentration of LC3 I (though not significantly; Fig. 5A). This indicates a potent inhibitory effect on autophagic accumulation. In a similar time frame, spautin-1 did not modify quinacrine uptake parameters (transport experiment over a 4-hr period, Fig. 5B), indicating that the protracted autophagic accumulation is a distal event relative to quinacrine transport.

**Figure 6:**
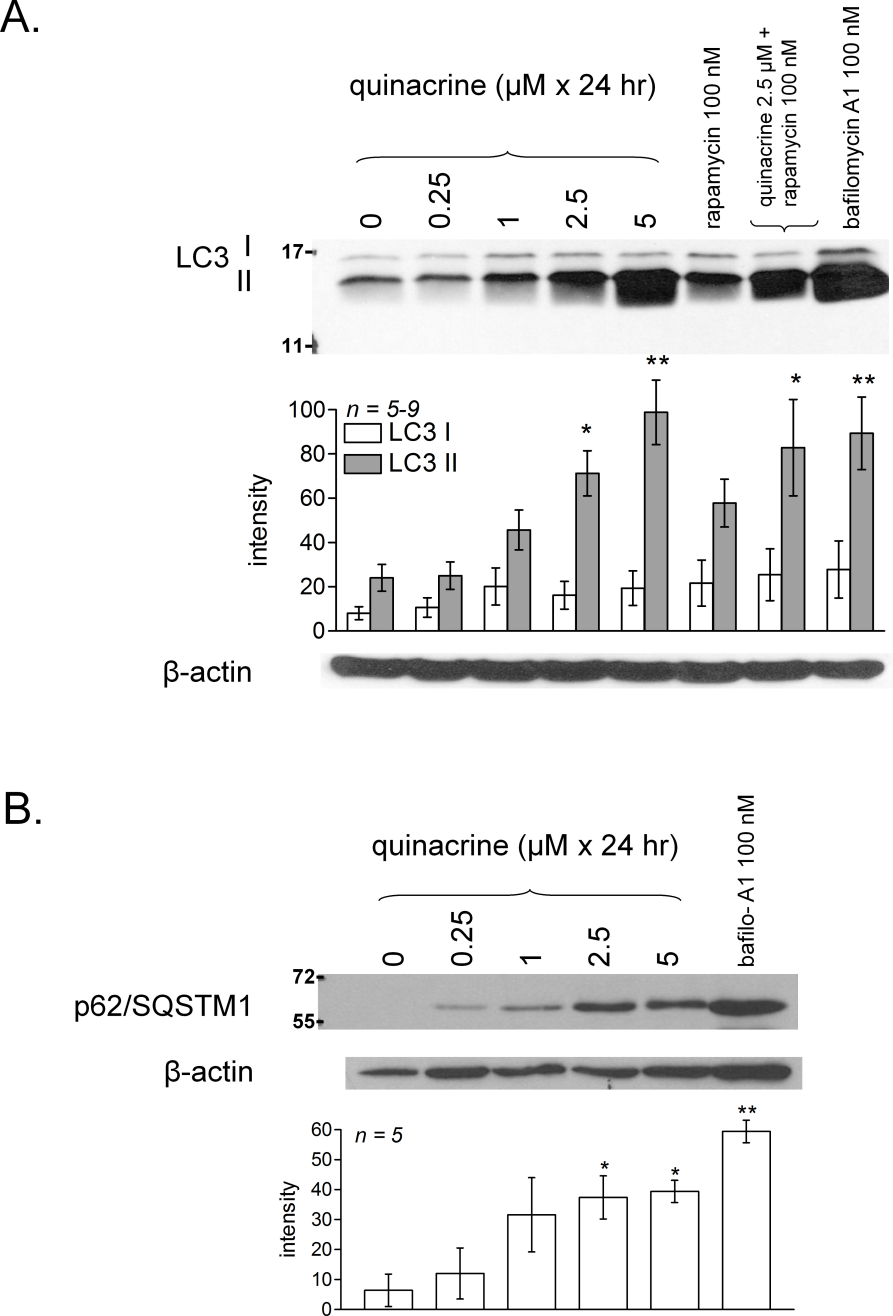
Characterization of quinacrine-induced autophagic accumulation in murine fibroblasts and comparison with other treatments. Effect of 24-hr treatments with quinacrine or other drugs on the accumulation of LC3 (A) and p62/SQSTM1 (B) in the total cell extract (immunoblots). In (A) and (B), histograms represent average densitometry values of immunoblots for replicated experiments (replicate number indicated by *n*). LC3 I values (A) did not vary significantly between groups, but quantities of LC3 II (A) and p62 (B) were heterogeneous (ANOVA: *P* < 10^−4^ and 0.001, respectively). Comparison of values with the common control values for each protein: ^∗^ *P* < 0.05; ^∗∗^ *P* < 0.01 (Dunnett’s test).

Dermal fibroblasts obtained from wild type C57BL/6 mice were cultured for 24 h in the presence of quinacrine to more closely mimic the time schedule of *in vivo* drug administration. LC3 II accumulation occurred in a concentration-dependent manner, with statistically significant effects from 2.5 µM (Fig. 6A). This type of response to “lysosomotropic” drugs may be the inhibition of the “autophagic flux,” meaning that the turnover of the basal level of autophagy and of its associated proteins is inhibited, without triggering the cell starvation signalling that is coupled to increased autophagy (Li, Chen & Gibson, 2013). If the buffering of acidic organelle pH is sufficient to explain LC3 II accumulation (Funakoshi et al., 2013), it should be reproduced by the V-ATPase inhibitor bafilomycin, a hypothesis that has been verified in the present system (Fig. 6A). Rapamycin had only a slight effect after 24-h treatment, and it was not additive with that of quinacrine (2.5 µM). p62/SQSTM1 is both a protein that plays a role in macroautophagy and an indicator of the autophagic flux (see ‘Introduction’). Over a 24-hr period, quinacrine (≥2.5 µM) or bafilomycin A1 induced a significant accumulation of p62/SQSTM1 over a very low basal level in control fibroblasts (Fig. 6B).

### Lysosomogenesis in quinacrine-treated murine fibroblasts

**Figure 7:**
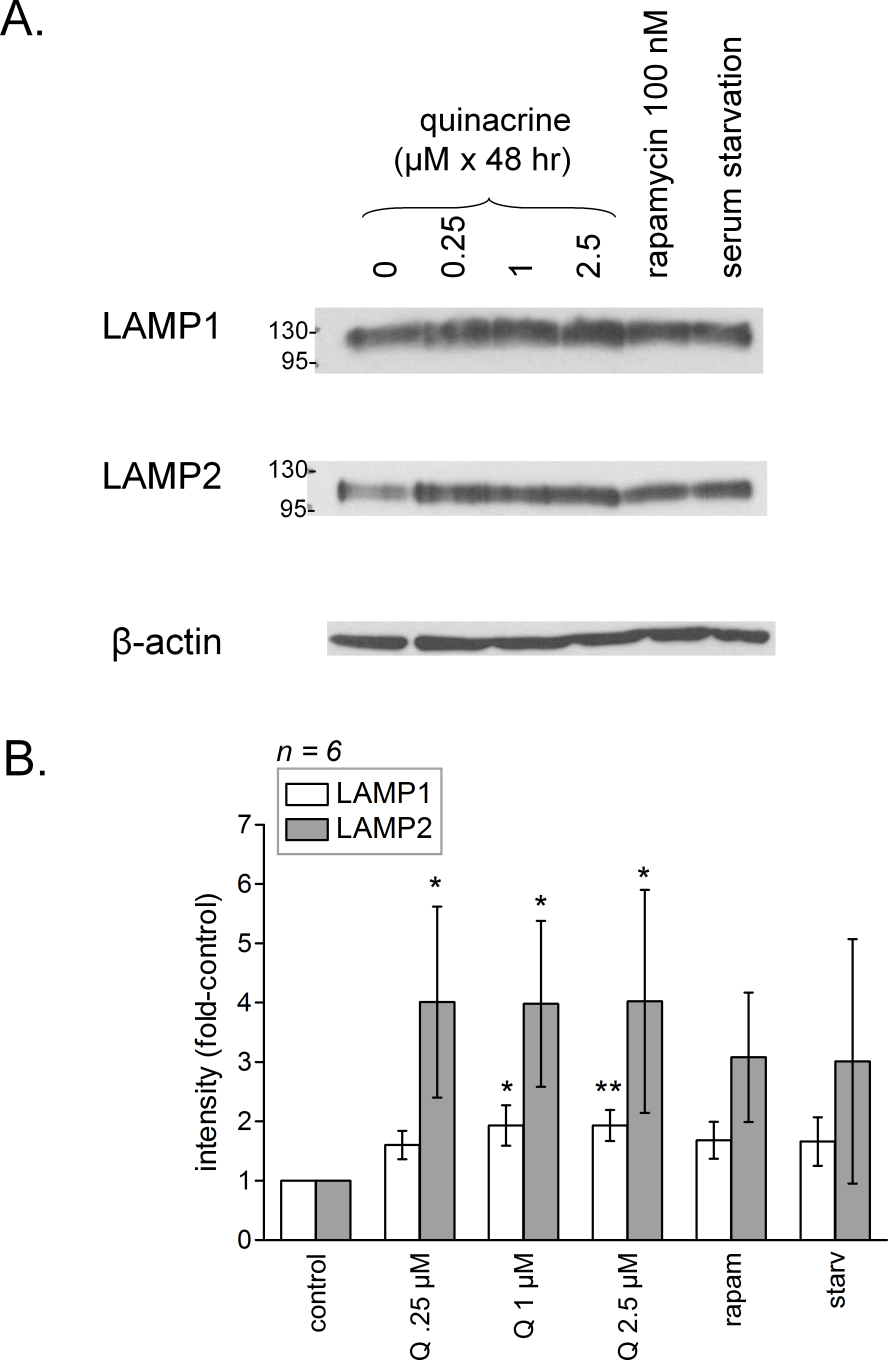
(A) Prolonged (48 h) effect of quinacrine and other treatments on feed-back lysosomal genesis in C57BL/6 mouse dermal fibroblasts as assessed using immunoblotting of LAMP1 and LAMP2. (B) Average densitometry values of immunoblots for replicated experiments. LAMP1 and LAMP2 quantities significantly vary between groups (*P* < 0.05, Kruskal–Wallis test). Comparison of values with the common control for each protein: ^∗^ *P* < 0.05; ^∗∗^ *P* < 0.01 (Dunn’s multiple comparison test).

**Figure 8:**
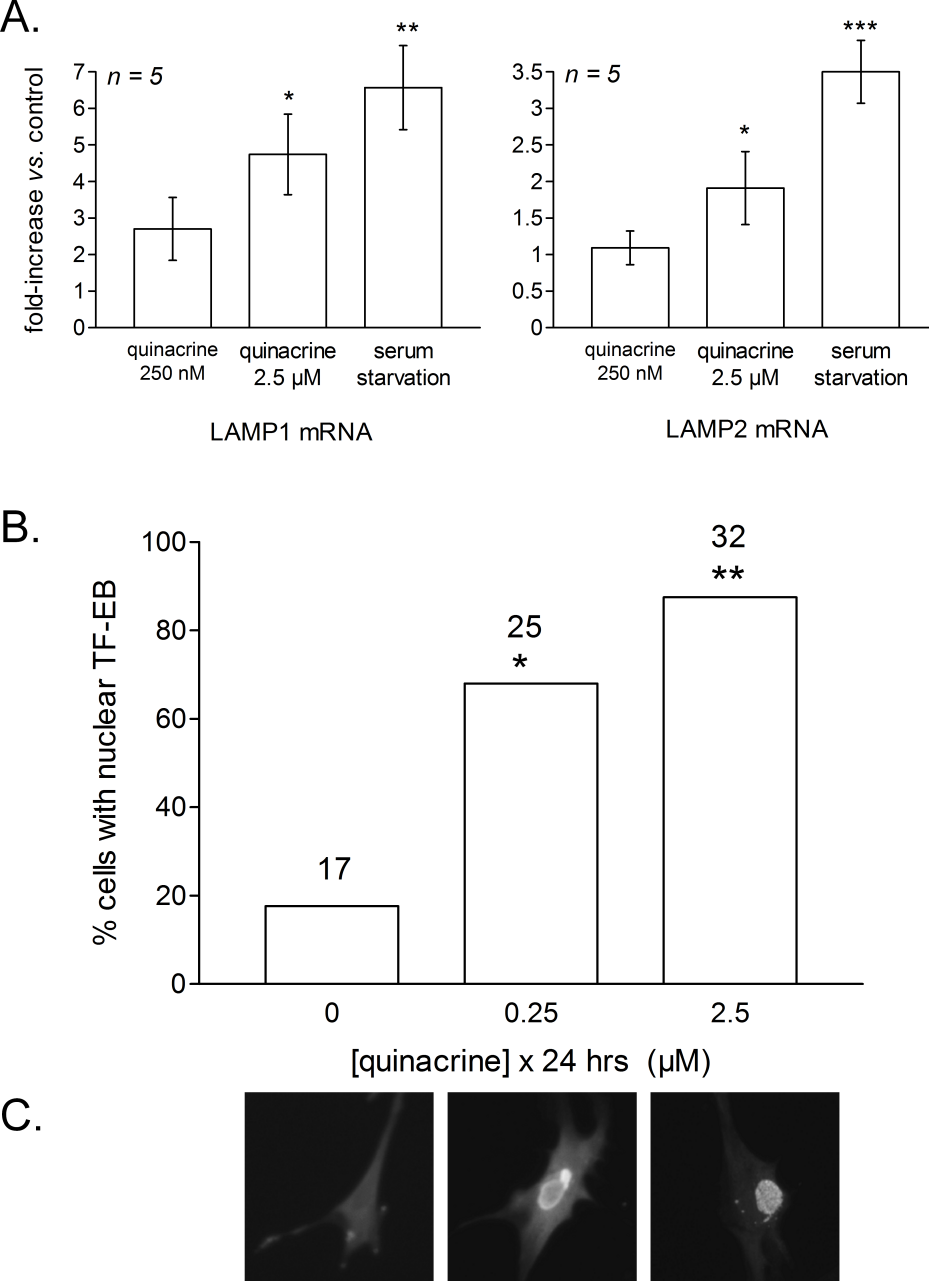
RT-PCR determination of LAMP1 and LAMP2 mRNA levels in mouse fibroblasts treated as indicated for 24 h. (A) The ratio LAMP/GAPDH has been normalized to 1 in each control cells well of the 5 series of wells and the results are expressed as fold increase vs. controls. Kruskal–Wallis test indicated that both LAMP1 and LAMP2 values were heterogeneous (*P* < 0.01 for each set). ^∗^ *P* < 0.05; ^∗∗^ *P* < 0.01; ^∗∗∗^ *P* < 0.001 vs. controls (Dunn’s multiple comparison test for selected pairs). (B, C) Quinacrine-induced TFEB nuclear translocation in mouse fibroblasts transiently expressing the myc-tagged protein (direct immunofluorescence in fixed and permeabilized cells). Sample photomicrographs are shown in (C). Histograms represent the proportion of cells with a definite nuclear labeling, indicating TFEB nuclear translocation. The numbers of evaluated cells (positive for any staining) in three separate experiments are indicated above bars. *χ*^2^ statistics: ^∗^ *P* < 0.01, ^∗∗^ *P* < 0.001 vs. the control cells.

Feed-back lysosomal genesis may be a consequence of inhibiting or buffering the acidification of V-ATPase containing organelles (see ‘Introduction’). As judged by the cell abundance of LAMP1 and LAMP2, a 48-hr quinacrine treatment (≥1 or 0.25 µM, respectively) significantly increased the mass of late endosomal/lysosomal compartments where these proteins are found (Fig. 7). Serum starvation, a known stimulant of autophagy (Li, Chen & Gibson, 2013), and rapamycin were less active in this respect (effects did not reach statistical significance). Only serum starvation significantly upregulated LAMP1 if the treatments are limited to 24 h (data not shown). RT-PCR performed 24 h after quinacrine stimulation or serum starvation indicated that both types of stimuli increased LAMP1 and LAMP2 mRNAs in fibroblasts, the drug having a significant effect at 2.5 µM but not 250 nM (Fig. 8A).

The transcription factor TFEB controls the expression of LAMP1, LAMP2, V-ATPase subunits and the expression of hundreds of other genes encoding proteins present in lysosomes (Sardiello et al., 2009; Peña-Llopis et al., 2011). Fibroblasts transiently expressing recombinant TFEB tagged with the myc epitope were submitted to immunofluorescence using an anti-myc antibody (Figs. 8B and 8C). In resting cells, TFEB was generally cytosolic but 24 h-treatments with quinacrine increased the frequency of nuclear labeling (significantly at both 0.25 and 2.5 µM concentration levels; Figs. 8B and 8C).

**Figure 9:**
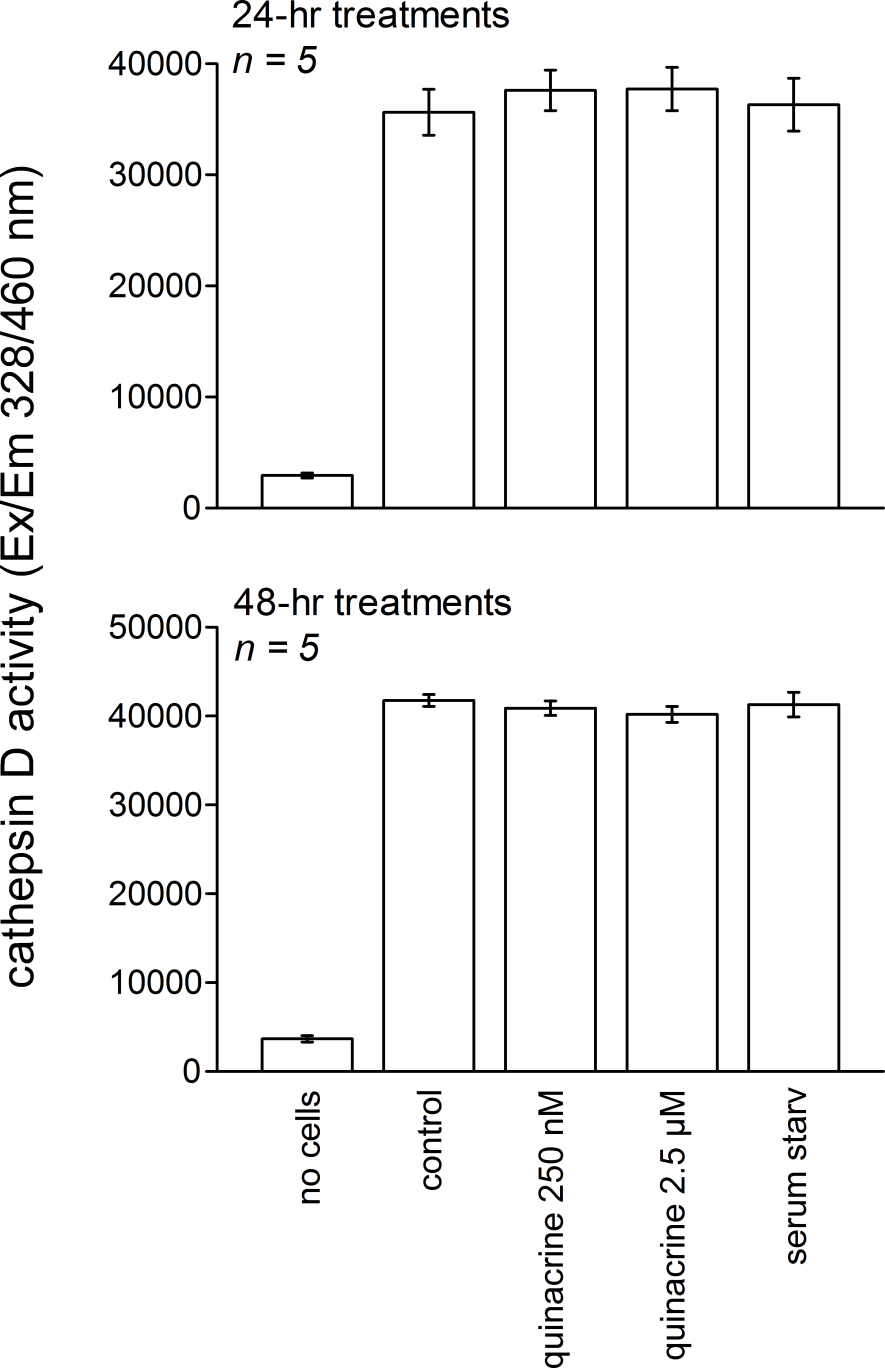
Cathepsin D activity in extracts of 10^6^ mouse fibroblasts treated for 24 or 48 h as indicated. ANOVA did not support that the activity in cells extracts were significantly different from each other.

Whether quinacrine increases the activity of a lysosomal hydrolase, cathepsin D, has been examined in mouse fibroblasts treated for 24 or 48 h (Fig. 9). Quinacrine treatment or serum starvation failed to modify the enzymatic activity vs. that recorded in control cells. The latter was much higher than a blank reading (without cell extract).

### Ancillary experiments related to the investigation of the modulatory effect of transporters on quinacrine transport

CEM cells are a line of human leukemic lymphoblasts; a subclone termed CEM-VLB has been selected *in vitro* for high resistance to vinblastine and overexpresses a glycoprotein later termed PGP (Beck, Mueller & Tanzer, 1979). Firstly, the exclusive expression of PGP in CEM-VLB cells was validated using immunoblotting of total cell extracts; CEM cells did not express PGP (Fig. S1 and Supplemental Information 1). Secondly, the uptake of doxorubicin (2.5 µM, 30 min) was monitored in both CEM and CEM-VLB cells, as the drug is known to be actively extruded via PGP at the plasma membrane level (Coley, Twentyman & Workman, 1993) and, as a DNA-binding molecule, is essentially found in nuclei. Indeed, the PGP-expressing cell clone CEM-VLB has a much decreased nuclear staining induced by doxorubicin (2.5 µM, Fig. S2, (A) microscopy; (B) cytofluorometry). Bafilomycin A1 co-treatment did not affect doxorubicin uptake in either cell type, but the PGP inhibitor elacridar significantly increased doxorubicin retention only in CEM-VLB cells. This gain of function in elacridar-treated CEM-VLB cells is consistent with the presence of functional PGP. Similar experiments compared the uptake of quinacrine between CEM and CEM-VLB cells (Fig. S3). Quinacrine uptake, mainly granular at or below 10 µM, was similar in the 2 cell types and suppressed by bafilomycin A1 treatment. However, the highest tested quinacrine concentration (30 µM) was associated with a strong nuclear labeling that was not abated by bafilomycin co-treatment in either cell subline (microscopy), consistent with the known affinity of the drug for DNA. The transport of the drug, quantified using cytofluorometry using the green fluorescence over the 0–30 µM concentration range, was similar in the two cell subtypes. PGP-mediated extrusion of quinacrine, if it happens at all at the tested concentration range, does not significantly modulate the vacuolar or nuclear concentration of the drug.

Additional experiments involved HEK 293a cells, that readily express transgenes following expression vector transfection and do not express the PGP protein (Fig. S1). As in several other cell types, incubation of HEK 293a cells with quinacrine (1 or 2.5 µM) determined an intense fluorescent labeling of perinuclear granules; some uniform green nuclear staining was also observed at the higher concentration without apparent cell damage (Fig. S4A). Cell co-treatment with the V-ATPase inhibitor bafilomycin A1 abolished the granular uptake of quinacrine, but not the nuclear staining, consistent with drug concentration in acidic vacuoles and low-affinity direct binding to DNA. Doxorubicin-associated red fluorescence stained the cell nuclei in a concentration-dependent manner (1–5 µM) and in a manner unaffected by bafilomycin A1 co-treatment, consistent with its direct binding to DNA (Fig. S4B). However, low intensity and bafilomycin-sensitive uptake of doxorubicin was also seen in perinuclear granules at the higher drug concentration levels, showing the inverse relationship of affinities for subcellular compartments vs. quinacrine.

The transport of quinacrine in total HEK 293a cells over a 30-min incubation period, as measured in total cell extracts, could be modeled as a hyperbolic curve with the following parameters: *V*_max_ 14.1 ± 2.3 nmol/well/30 min, *K*_M_14.1 ± 2.5 µM (Fig. S5). In cells co-treated with bafilomycin A1, the magnitude of quinacrine uptake was drastically, but not completely abated (*V*_max_ 2.3 ± 0.5 nmol/well/30 min). The residual quinacrine uptake may correspond to nuclear staining as observed in microscopic experiments. As in murine fibroblasts, quinacrine treatment determined the accumulation of the cleaved and lipidated autophagic effector, LC3 II in HEK 293a cells (4 h-treatments; Fig. S6); bafilomycin A1 was also active in this respect, but the effect of rapamycin did not reach statistical significance. Again, co-treatment with spautin-1 significantly reduced the effect of quinacrine and bafilomycin A1 (Fig. S6). Corroborating studies in fibroblasts, spautin-1 cotreatment did not modify quinacrine transport into HEK 293a cells over 4 h (Fig. S7 A: microscopy; B: transport as a function of quinacrine concentration), suggesting that autophagy is a secondary and distal response to cell concentration of the amine.

Myc-tagged construction of human OCT-1 to -3 were encoded in vectors that were transfected into HEK 293a cells (Fig. S8). The anti-myc monoclonal antibody 4A6 revealed double bands of protein (∼55 and 85 kDa) for each of the construction in the extracts of cells transiently transfected with the vectors, as reported by the manufacturer. Immunofluorescence of transfected cells evidenced the expression of the transgenes at various levels, including plasma membranes, and the uptake of a relatively low concentration (100 nM) of [^3^H]MPP^+^, a promiscuous OCT-1 to -3 substrate (Nies et al., 2011), was significantly greater in OCT-expressing cells than that of mock-transfected cells (Fig. S8 and Supplemental Information 1). The OCT inhibitor decynium-22 slightly abated quinacrine transport in HEK 293a cells (Fig. S9). However, OCT-expressing cells did not evidence altered quinacrine transport parameters (micromolar concentration range; Fig. S9B: *V*_max_ and *K*_M_ values similar to those of mock-transfected cells).

### Quinacrine distribution in mouse tissues

**Figure 10:**
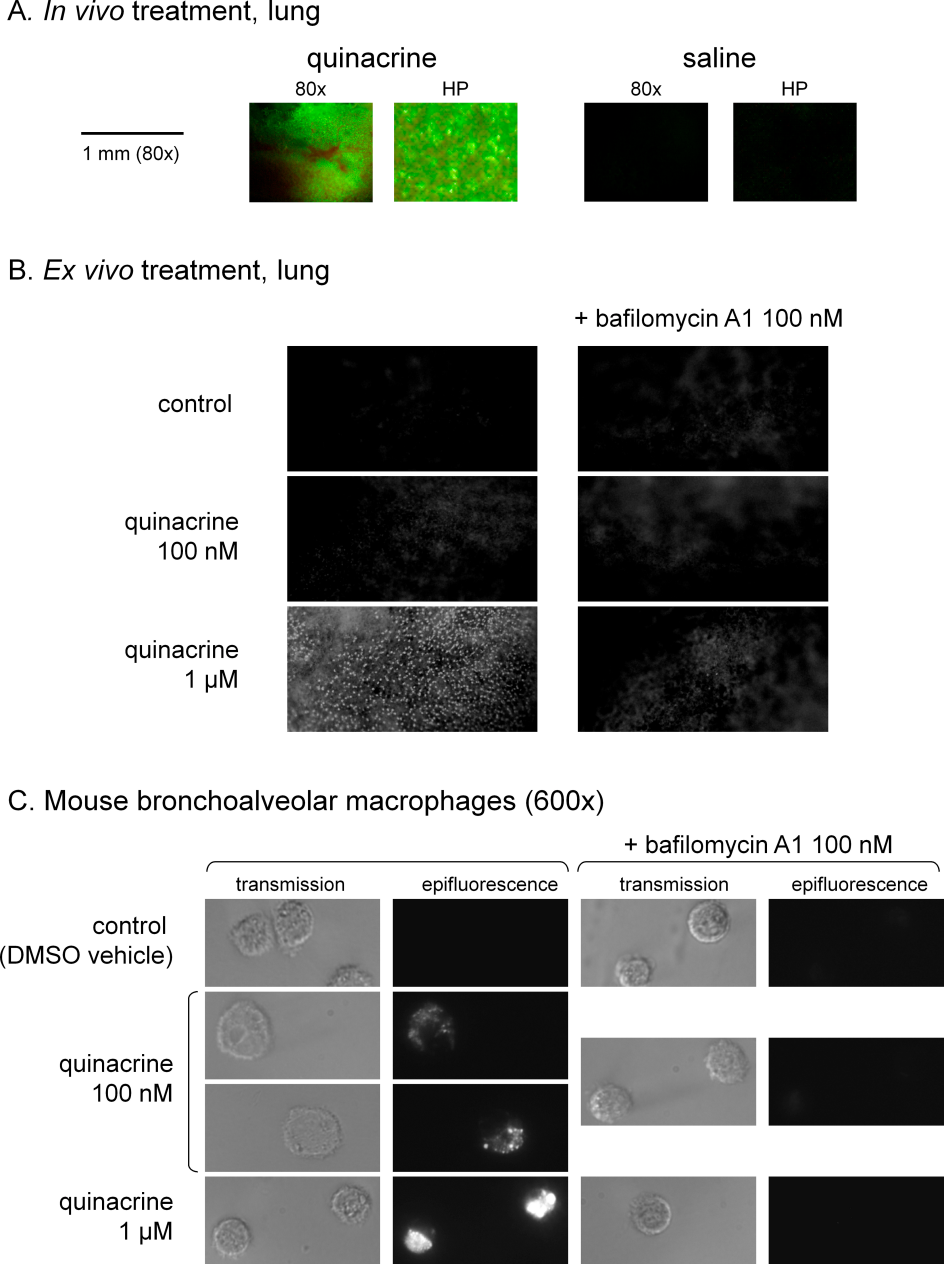
Tropism of quinacrine for mouse lungs. (A) Lung slices of mice treated *in vivo* with quinacrine (40 mg/kg/day) over 48 h viewed using fluorescence dissection microscopy (magnification either 80× or high power, HP). The organs of saline-treated animals are shown to evidence the level of autofluorescence. (B) *Ex vivo* staining of fresh lung slices with quinacrine, with optional bafilomycin A1 co-treatment. The slices were incubated in Hank’s balanced salt solution at 37 °C, to which drugs were added, for a period of 30 min. Magnification 80×. (C) *Ex vivo* staining of mouse bronchoalveolar macrophages with quinacrine, with optional bafilomycin A1 co-treatment. The cells were plated for 1 h, then treated with drugs for 4 h before observation (magnification 600×).

Quinacrine administration over 48 h had no observable effect on live mice (behavior, weight). In lungs dissected from quinacrine-treated mice (40 mg/kg/day), the green fluorescence of the drug could be observed as a discontinuous dot pattern (Fig. 10A; negligible autofluorescence judged from organs of saline-treated mice). Fresh slices of lungs from untreated mice incubated for 30 min at 37 °C take up quinacrine applied *in vitro* (1 µM in Hank’s balanced salt solution) under the form of discrete bright dots dispersed in the alveolar structure (Fig. 10B). Bafilomycin A1 co-treatment strongly abated this uptake that possibly concerns alveloar macrophages. A study of the latter cells, obtained after bronchoalveolar lavage, supports the high affinity uptake of quinacrine (0.1 or 1 µM, 4 h), based on microscopy (Fig. 10C).

A quantitative approach based on fluorometry was applied to measure quinacrine uptake in the organ extracts from separate groups of mice submitted to the same *in vivo* treatments. Consistent with previous reports (Ehsanian, Van Waes & Feller, 2011), the liver and spleen concentrated large quantities of the drug, but the brain almost none, while other organs were intermediary (Fig. S10). Residual blood content in organs is not expected to distort these tissues distributions because mouse red blood cells do not take up quinacrine *in vitro* (Fig. S11), consistent with the absence of lysosomes in mature erythrocytes (Kornfeld & Gregory, 1969). Further, quinacrine is efficiently removed from the extracellular fluid, including blood plasma (Ehsanian, Van Waes & Feller, 2011), by tissue uptake *in vivo*.

**Figure 11:**
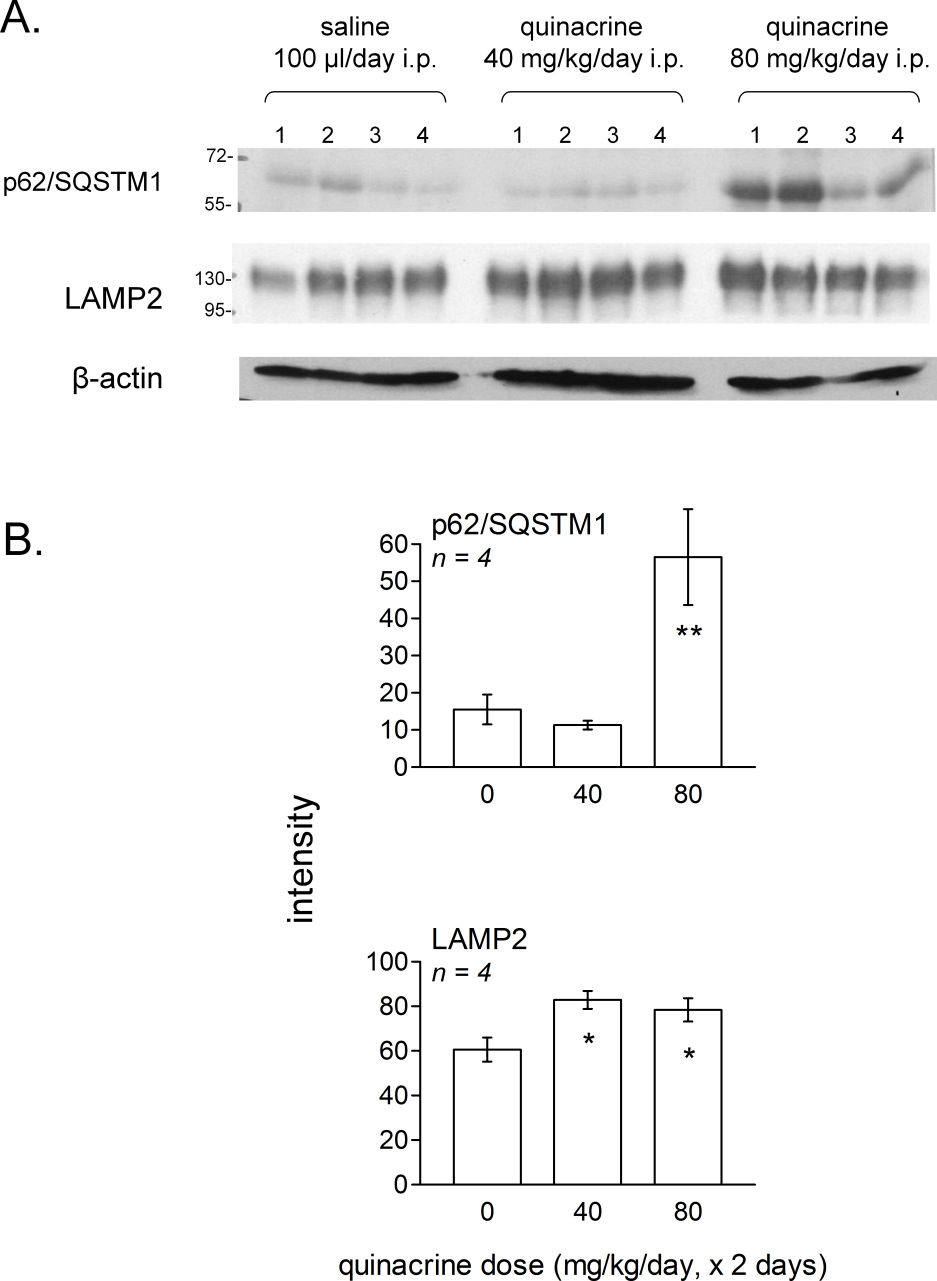
Effect of *in vivo* treatment with quinacrine on autophagic and lysosomogenesis markers as assessed by immunoblotting of lung tissue extracts. (A) Groups of 4 mice were treated twice at 24-hr interval, as indicated, and euthanized 24 h after the last dose. Numbers 1–4 in each experimental group represent extracts derived from each experimental animal. (B) averaged quantities of p62/SQSTM1 and LAMP2 were heterogeneous between groups (ANOVA: *P* < 0.01 and 0.05, respectively). Comparison of values with the common control group for each protein: ^∗^ *P* < 0.05; ^∗∗^ *P* < 0.01 (Dunnett’s test).

To extend *in vivo* the signaling experiments to the most discriminative markers identified using murine fibroblasts, we selected the lung, an extra-abdominal organ remote from the peritoneal site of drug administration. Lungs of mice treated with quinacrine for 2 days (40 or 80 mg/kg/day) or with the saline vehicle were homogeneized and the extracts were submitted to immunoblotting. In lungs, a dose-dependent significant accumulation of p62/SQSTM1 was observed (significant at 80 mg/kg/day; Fig. 11). Thus, autophagic flux was inhibited 24 h after the last dosing at this level. The lysosomal glycoprotein LAMP2 was modestly, but significantly increased in lungs of mice treated with either dose levels.

## Discussion

**Figure 12:**
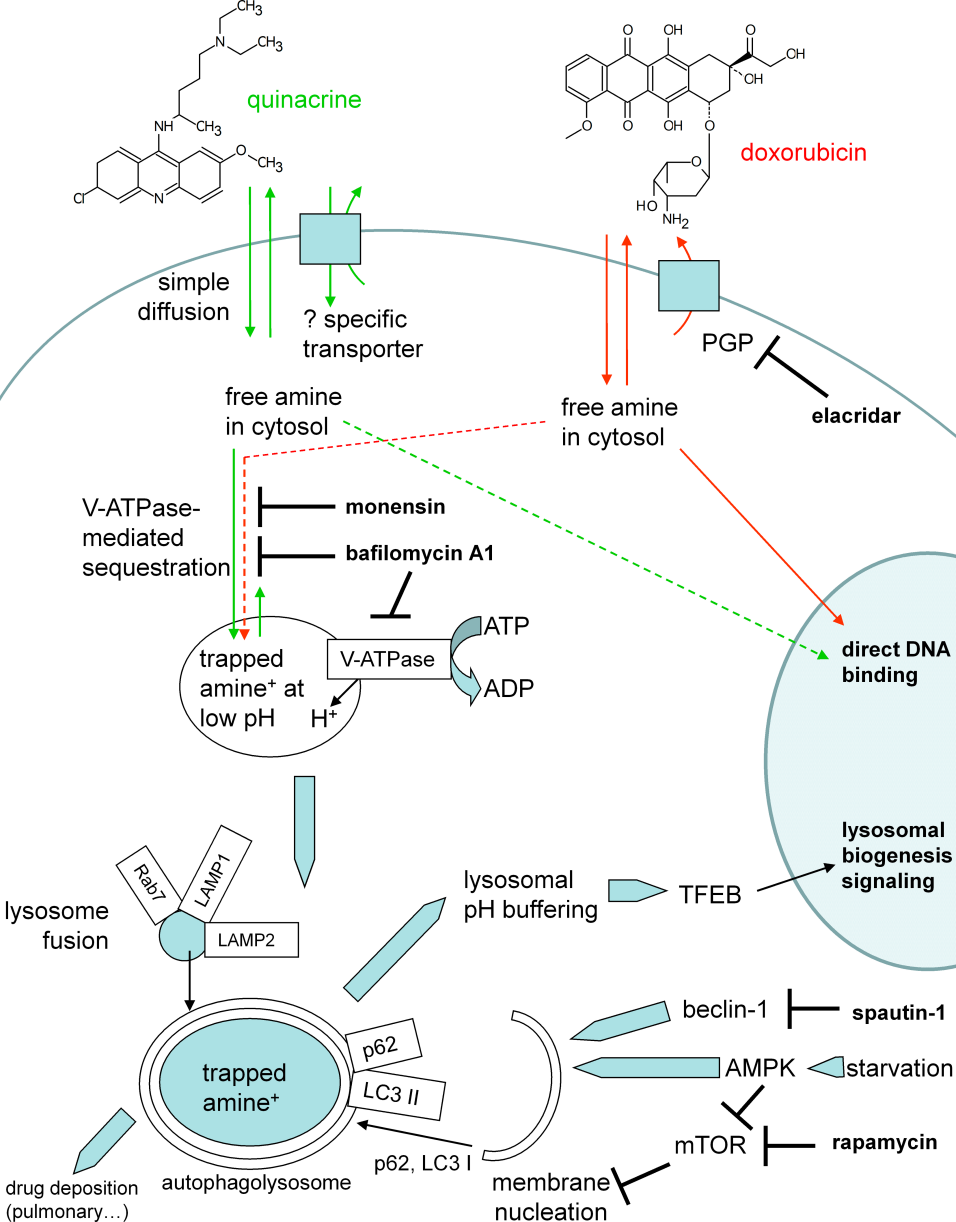
Schematic representation of cell responses to quinacrine. The mode of action of other drugs or interventions used in the study is also indicated.

Quinacrine has 2 protonable functions (pK_a_ 10.3, 7.7) and high lipophilicity (logP 5.67); considerations relevant for its use *in vivo* are its low toxicity (nonmutagenic although it can bind to DNA at high concentrations) and the fact that its major metabolite, a desethyl secondary amine (Huang et al., 2006), keeps its tropism for acidic organelles, high lipophilicity and fluorescence (schematic representation, Fig. 12). The specific inhibitor of all mammalian V-ATPase isoforms, bafilomycin A1, extensively reduced the > thousand-fold concentration of this drug in murine fibroblasts (Figs. 1, 3 and 4); this also applied to HEK 293a cells (Fig. S5). Further, the H^+^ ionophore monensin also prevented the uptake of quinacrine in fibroblasts (Fig. 4A), confirming the ion trapping model via an alternate approach. While genetic inactivation of key functional V-ATPase subunits determines embryonic lethality in the mouse (Inoue et al., 1999), and pharmacologic inhibition of V-ATPase *in vivo* seems to be poorly tolerated (Niikura, Takeshita & Takano, 2005), experiments made using fresh slices of mouse lungs and bronchoalveolar macrophages treated with bafilomycin A1 supported the general role of V-ATPase in quinacrine uptake in organs. The partition of quinacrine into vacuolar phospholipids is a possible explanation for the slow reversibility of quinacrine uptake upon cell washing (Fig. 3A). A more hydrophilic substituted trimethylamine, procainamide, was rapidly released following cell washing in a similarly designed experiment (Morissette, Lodge & Marceau, 2008). Another possibility concerns precipitation of a highly concentrated solution in vacuoles. The alternate lipophilic drug, clofamizine, forms crystals in inclusions of macrophages in various organs of chronically dosed mice (Baik et al., 2013). CEM and CEM-VLB cells exhibited bafilomycin-independent nuclear staining following treatment with 30 µM quinacrine; in addition, HEK 293a cells treated with 2.5 µM quinacrine, some nuclear staining was evident only in bafilomycin A1-treated cells, as if inhibition of drug concentration in acidic organelles determined an intracellular redistribution of the drug. Both observations are consistent with the low-affinity binding of quinacrine to DNA. Doxorubicin’s relative affinities for these two targets are opposite: the nuclear staining occurs at lower concentration than that producing vacuolar concentration for this alternate cationic drug which possesses the teracene planar structure instead of the acridine (Fig. S4; Fig. 12). Other anthracyclines may be almost equally drawn to both compartements, like daunorubicin (Ouar et al., 2003). Co-localization studies in murine fibroblasts recapitulated previous studies of cells with cationic drug reservoirs (Morissette, Lodge & Marceau, 2008; Marceau et al., 2009; Bawolak, Morissette & Marceau, 2010): most quinacrine-concentrating enlarged vacuoles also contained the late endosome/lysosome markers Rab7 and LAMP1 (Fig. 2). Co-localisation with the early endosome marker Rab5 was occasionally seen, especially if the constitutively active form of Rab5 was expressed (Fig. 2). This is compatible with the known expression of V-ATPase in diverse organelles.

The ion trapping model explains drug concentration and retention from the quasi-neutral cytosol to the acidic organelles (De Duve et al., 1974), but the apparent selectivity in tissue reservoir formation is intriguing (Fig. 10, Fig. S10). A high density of macrophages in some organs explains the concentration of quinacrine could be invoked for the lungs, spleen and liver. Quinacrine fluorescence was also observed in a tumor formed by a transplanted human glioma cell line in nude mice that were treated with 50 mg/kg of the drug (Golden et al., 2015). Theoretically, individual cell types may possess uptake or extrusion mechanisms that modulate quinacrine access to their cytosol (Fig. 12). Based on previous literature (see ‘Introduction’), we addressed the role of PGP and OCT-1 to -3 using cellular models (expression of recombinant transporters or use of a number of transport inhibitors). In the low micromolar range used for *in vitro* experiments, there is no unequivocal evidence of quinacrine transport by these plasma membrane macromolecules (Fig. 4, Figs. S1–S4 and S8–S9). Reports concerning quinacrine transport or binding to solute carrier transporters deal with vastly superior concentrations, usually above 50 µM (Sweet & Pritchard, 1999). Other possible explanations for tissue selectivity include: a role for alternate transporters not tested or covered by the promiscuous inhibitors, a V-ATPase-independent nuclear uptake in some cell types, a larger cell reservoir of acidic organelles or a higher capacity for feed-back lysosomogenesis in some cell types, differential effects of V-ATPase isoforms on cation trapping and more effective pinocytosis, perhaps explaining the high affinity uptake of basic drugs in mature phagocytes (Roy et al., 2013).

Independent of the therapeutic class, accumulation of lipophilic cationic drugs may determine cell signaling responses that are the consequence of lysosomal buffering and impairment: macroautophagic accumulation and lysosomogenesis (see ‘Introduction’). These predictions were verified in murine models, including lung tissue from quinacrine-treated mice (Fig. 11). Autophagic accumulation is secondary to and does not affect quinacrine transport, based on the effect of spautin-1. TFEB translocation to cell nuclei and the upregulation of LAMP1 and -2 mRNA concentrations were effects of quinacrine on cultured fibroblasts (Fig. 8) that preceded the upregulation of lysosomal proteins LAMP1 and -2 (Fig. 7). Interestingly, TFEB also controls the expression of autophagy genes (Settembre & Ballabio, 2014). The activity of lysosomal cathepsin D was not altered in fibroblasts treated with quinacrine (Fig. 9); this apparent paradox may be related to the maturation of the cathepsin proenzyme, a cleavage dependent of the acidic environment of mature lysosomes (Liu et al., 2014). Thus, in cells in which autophagosomes of dubious functionality have accumulated, quinacrine-induced lysosomogenesis is hypothetically part of a finely regulated feedback that keeps the lysosomal hydrolase activity constant. Future research orientations include the clinical detection of biomarkers of cationic drug reservoir formation in peripheral blood leukocytes, such as autophagic and lysosomal proteins and, perhaps, immature lysosomal hydrolases (Liu et al., 2014). The basis for preferential tissue distribution of amines also remains to be elucidated.

The tissue distribution of quinacrine in dosed mice varies considerably from one organ to the other, but no mechanism modulating the cellular uptake of micromolar quinacrine concentration levels has been isolated. As shown *in vitro* and *in vivo*, V-ATPase-mediated cation sequestration is associated, above a certain threshold, to autophagic accumulation and feed-back lysosomogenesis in murine models.

## Supplemental Information

10.7717/peerj.1314/supp-1 Supplemental Information 1 Supplemental methods

10.7717/peerj.1314/supp-2 Figure S1 Verification of PGP expression in 3 human cell types, HEK 293a, CEM and CEM-VLB, using immunoblot of total cell extracts Parallel immunoblotting for *β*-actin was performed to document equal loading of tracks. Representative of three separate experiments.

10.7717/peerj.1314/supp-3 Figure S2 Verification of PGP function in CEM-VLB cells using the fluorescence of doxorubicin, a known extrusion substrate Cells were treated at 37 °C for 30 min with doxorubicin (2.5 µM), with optional co-treatment applied 15 min before doxorubinin (bafilomycin A1 100 nM or elacridar 5 µM). (A) Transmission and epifluorescence microscopy of representative cells (600×) are represented side by side with cytofluorometric distribution of the drug-associated fluorescence. (B) Mean cell red fluorescence in replicated experiments. The effect of drugs on doxorubicin uptake was tested with ANOVA for CEM cells (non-significant) or CEM-VLB cells (*P* < 0.05). Only the co-treatment with the PGP inhibitor elacridar significantly changed the uptake of doxorubicin into CEM-VLB cells (*P* < 0.05, Dunnett’s test).

10.7717/peerj.1314/supp-4 Figure S3 Uptake of quinacrine (2.5 µM, 30 min) in CEM or CEM-VLB cells, with optional co-treatment applied 15 min before (bafilomycin A1 100 nM) (A) Transmission and epifluorescence microscopy of representative cells (600×). (B) Cytofluorometric distribution of the drug-associated fluorescence in the 2 cell subtypes.

10.7717/peerj.1314/supp-5 Figure S4 Morphological evidence of fluorescent drug uptake in HEK 293a cells Morphological evidence of fluorescent drug uptake (A, quinacrine; B, doxorubicin; other treatments as indicated) in perinuclear vacuoles and/or nuclei of HEK 293a cells and effect of co-treatment with the V-ATPase inhibitor bafilomycin A1 on the drug uptake and subcellular distribution.

10.7717/peerj.1314/supp-6 Figure S5 Uptake of quinacrine into HEK 293a Uptake of quinacrine into HEK 293a cells as measured using extract-associated fluorescence: effect of quinacrine concentration on the uptake during a 30-min period as modified by an optional bafilomycin A1 co-treatment.

10.7717/peerj.1314/supp-7 Figure S6 Evidence of macroautophagic accumulation in HEK 293a cells treated for 4 h with quinacrine and other drugs, as indicated The figure includes a representative immunoblot of total cell extract revealed with anti-LC3 antibodies, a matched immunoblot for *β*-actin to document equal loading of tracks and the densitometric evaluation of LC3 I and II cell contents in replicated experiments and reported as histograms. The effect of the autophagic inhibitor spautin-1 was validated. LC3 I concentration between groups did not significantly differ (ANOVA). LC3 II values were heterogeneous (ANOVA *P* < 10^−4^; Bonferroni multiple comparison test for selected pairs: ^∗^ *P* < 0.01, ^∗∗^ *P* < 0.001 vs. control; ^†^ *P* < 0.01, ^††^ *P* < 0.001 vs. same treatment without spautin-1).

10.7717/peerj.1314/supp-8 Figure S7 Lack of effect of spautin-1 co-treatment on the uptake of quinacrine measured over a 4-hr incubation period into HEK 293a cells (A) Microscopy. Presentation as in Fig. S4. (B) Transport evaluated using fluorescence of cell extracts (presentation as in Fig. S5).

10.7717/peerj.1314/supp-9 Figure S8 Validation of the transient expression of myc-tagged OCT in HEK 293a cells (A) Immunoblot for the myc tag of the constructions. Representative of 2 experiments. (B) Direct immunofluorescence (AlexaFluor-488 conjugated anti-myc tag antibody) of fixed and permeabilized HEK 293a cells transiently expressing OCTs. (C) Uptake of MPP+ (100 nM) as influenced by the expression of OCTs. Results are expressed as fold of the values recorded in mock-transfected cells (absolute average value 283 ± 53 fmol/well). Values were significantly heterogeneous (ANOVA, *P* < 0.01). ^∗^ *P* < 0.01 vs. mock-transfected cells.

10.7717/peerj.1314/supp-10 Figure S9 Quinacrine transport in HEK 293a cells (A) Effect of the multivalent OCT inhibitor decynium-22 on the transport of quinacrine in total HEK 293a cell extracts. (B) Effect of overexpression of myc-tagged OCTs on the transport of quinacrine by transfected HEK 293a cells (expressed in absolute values, or as a percent of the maximal transport recorded at 30 µM; no significant differences were observed).

10.7717/peerj.1314/supp-11 Figure S10 Quinacrine-like fluorescence in extracts of mice dosed *in vivo* with either quinacrine (40 mg/kg/day i.p. for 2 days) or its saline vehicle The autofluorescence is quantified in the extracts of saline-treated animals and was compared to the fluorescence in organ extracts from drug-treated animals using Student’s *t* test (^∗^ *P* < 0.05; ^∗∗^ *P* < 0.01; ^∗∗∗^ *P* < 0.001).

10.7717/peerj.1314/supp-12 Figure S11 Uptake of quinacrine by mouse blood cell *in vitro* (60 min, 37 °C, 2.5 µM drug concentration) Quinacrine uptake, judged from fluorescence, is not present in red blood cells, but present in scattered leukocytes. Original magnification 600× (inserts at the right show some of the observed leukocyte morphologies).
