# Supplementary material for: Autophagic flux inhibition and lysosomogenesis ensuing cellular capture and retention of the cationic drug quinacrine in murine models
Source: PeerJ. 2015 Oct 6;3:e1314. doi: 10.7717/peerj.1314 (PMC4614855; doi:10.7717/peerj.1314)
Supplement: Supplemental Information 1 [file peerj-03-1314-s001.pdf]

Supplemental Methods

Cells and vectors

HEK 293a cells, originally obtained from Sigma-Aldrich, were cultured in DMEM supplemented with 10% fetal bovine serum and antibiotics and used in the majority of experiments. CEM cells are a line of human leukemic lymphoblasts; a subclone termed CEM-VLB has been selected in vitro for high resistance to vinblastine and overexpresses a glycoprotein later termed PGP (Baeck et al., 1979). Both cell types, non-adherent, were used to assess the effect of naturally expressed PGP on drug uptake. Vectors encoding the human sequences of selected transporters were obtained from OriGene Technologies (Rockville, MD; myc-tagged members of the SLC22A family organic cation transporters-1 to -3 (OCT-1 to -3), cat. no. RC211872, RC207921and RC218172). HEK 293a cells were transfected 48 hrs before transport experiments using a polyethyleneimine-based reagent as described (Morissette et al., 2008a). The coding sequence of OCT-3 was transferred into the empty vector pcDNA3.1 using general methods outlined elsewhere (Charest-Morin et al., 2013). The sequence corresponding to OCT3-DDK-Myc was amplified from the vector bought from origene using the following PCR primers: 5’-C GTT TAA ACG GGC CCT ATG CCC TCC TTC GAC GAG-3’ (sense) and 5’-TTG GTA CCG AGC TCG TTA AAC CTT ATC GTC GTC ATC CTT G-3’ (antisense). The PCR product was inserted in the XbaI/BamHI digestion product of the pcDNA3.1(-) vector using the Gibson Assembly MasterMix (NEB). This vector was validated by sequencing.

Cell transport

The uptake of quinacrine in HEK 293a cells was established using a variation of a technique previously applied to a different cell type (Marceau et al., 2009). A HEK 293a cells well containing 2 ml of culture medium in 12-well plates was the basis of experimental points. Test drugs were added to serum-containing culture medium according to various schemes and time frames. Quinacrine uptake at the end of the allowed incubation period (37°C, 5% CO_2) was determined by rapidly washing each cell well 3 times with 1 ml of phosphate-buffered saline, pH 7.4, at room temperature and dissolving the cells with 2.5 ml of 1 N NaOH. The recovered NaOH extracts were further diluted 1:10 in NaOH 1 N. Quinacrine was analyzed in the final dilution extract using an Aminco Bowman Series 2 luminescence spectrophotometer against a standard curve of the authentic drug dissolved in NaOH 1 N (excitation 414 nm, emission 501 nm).

Control fluorescence from extracts of untreated cells was systematically verified, of small magnitude and subtracted from experimental values.

[^{3}H]MPP$^+$ transport

Methyl-4-phenylpyridium acetate, N-[methyl-^{3}H] ([^{3}H]MPP$^+$), specific activity 80 Ci/mmol, was purchased from American Radiolabeled Chemicals, Inc. (St-Louis, MO). This drug is a promiscuous substrate for OCT-1, OCT-2 and OCT-3 (Nies et al., 2011). HEK 293a, grown in 24-well plates, were transfected with a vector encoding one of the OCTs or an empty vector. Then the cells were washed and each well, filled with 1.25 mL of warmed DMEM without serum supplemented with 100 nM [^{3}H]MMP$^+$. Cell were incubated at 37°C, in the presence of 5% CO_2, for 30 min, then rinced 3 times with 1.25 ml cold PBS and dissolved by a short treatment with 1 ml 0.1 N NaOH. The resulting suspensions were counted by scintillation.

Immunoblots

Lysates of cells were submitted to immunoblotting for LC3 after 24 h of treatment with quinacrine, rapamycin or bafilomycin A1 to determine whether cytosolic LC3 I (18 kDa) is converted into the macroautophagy effector, the lipidated and membrane bound LC3 II form (16 kDa) (Methods as in [31]). The expression of myc-tagged OCT constructions was verified in total HEK 293a cell extracts of transfected cells using the anti-myc monoclonal antibody (clone 4A6, dilution 1:1000). To ascertain the expression of PGP in CEM-VLB cells, extracts of equal numbers of non-adherent CEM-VLB and CEM cells were made as in Roy et al. (2013). The primary antibody was a mouse monoclonal anti-human PGP (clone JSB-1, Enzo Life Sciences, Farmingdale, NY) used at the 1:10 dilution. All extracts were migrated in SDS-PAGE, transferred, immunoblotted and revealed with the appropriate HRP-conjugated secondary antibodies (as in the main Methods).

*Cytofluorometry of CEM cell*s

Suspensions of CEM or CEM-VLB cells were treated with fluorescent and other drugs for 30-45 min in their regular culture medium, spinned and rinsed with PBS, resuspended in PBS and submitted to cytofluomoremetic analysis of the uptake of quinacrine (green fluorescence) or doxorubicin (red fluorescence), as described (Roy et al., 2013).

Uptake of quinacrine by mouse blood cells

Heparinized blood samples were obtained by cardiac puncture from deeply anesthetized mice. Ten µl samples were diluted with 90 µl of phosphate buffered saline (PBS) with or without quinacrine and the suspensions were incubated for 1 hr at 37°C. Cells were centrifuged, resuspended in 1 ml of PBS for rinsing, centrifuged again and resuspended in small volumes of PBS for microscopic observation on glass slides (transmission and green fluorescence).

Supplemental reference

Charest-Morin X, Fortin JP, Bawolak MT, Lodge R, Marceau F. 2013. Green fluorescent protein fused to peptide agonists of two dissimilar G protein-coupled receptors: novel ligands of the bradykinin B_2 (rhodopsin family) receptor and parathyroid hormone PTH_1 (secretin family) receptor. Pharmacological Research and Perspectives 1:e00004 DOI 10.1002/prp2.4.
