# Supplementary figures and images for: Autophagic flux inhibition and lysosomogenesis ensuing cellular capture and retention of the cationic drug quinacrine in murine models
Source: PeerJ. 2015 Oct 6;3:e1314. doi: 10.7717/peerj.1314 (PMC4614855; doi:10.7717/peerj.1314)

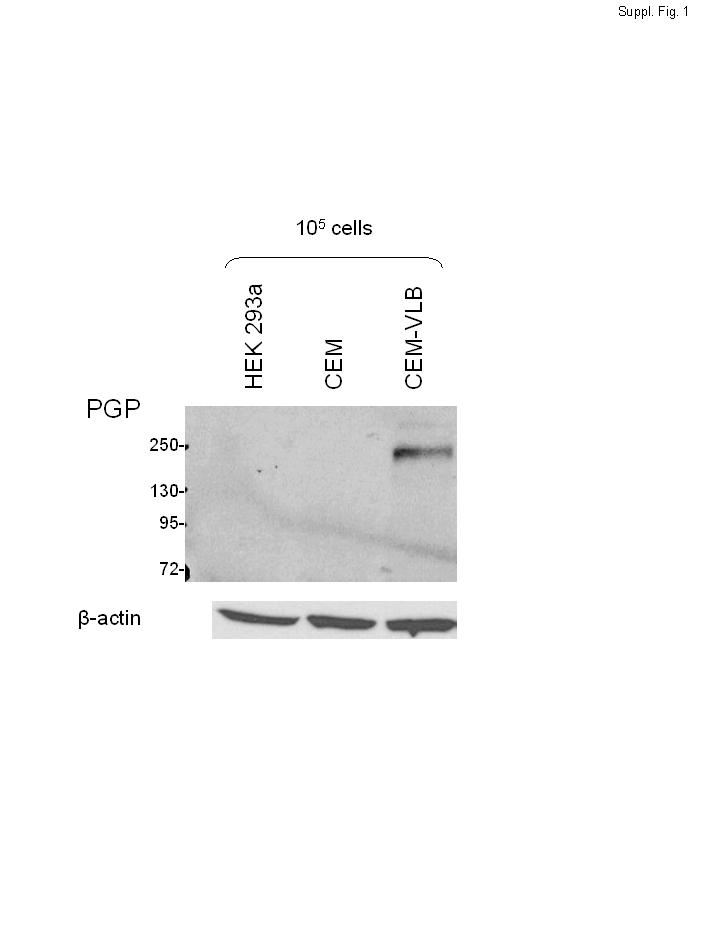

Supplement: Figure S1 — Parallel immunoblotting for β-actin was performed to document equal loading of tracks. Representative of three separate experiments. [file peerj-03-1314-s002.png]

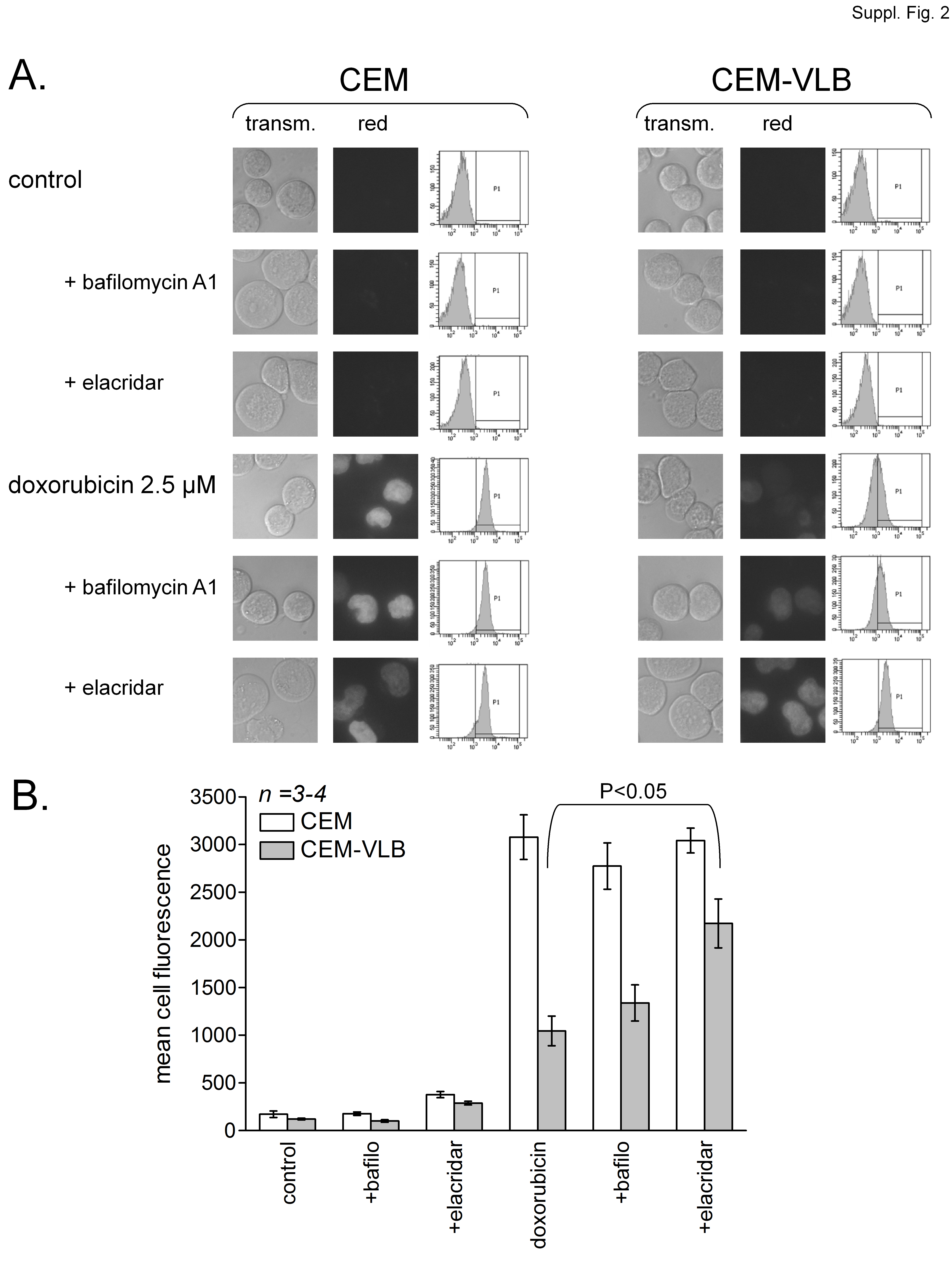

Supplement: Figure S2 — Cells were treated at 37 °C for 30 min with doxorubicin (2.5 µM), with optional co-treatment applied 15 min before doxorubinin (bafilomycin A1 100 nM or elacridar 5 µM). (A) Transmission and epifluorescence microscopy of representative cells (600×) are represented side by side with cytofluorometric distribution of the drug-associated fluorescence. (B) Mean cell red fluorescence in replicated experiments. The effect of drugs on doxorubicin uptake was tested with ANOVA for CEM cells (non-significant) or CEM-VLB cells (P < 0.05). Only the co-treatment with the PGP inhibitor elacridar significantly changed the uptake of doxorubicin into CEM-VLB cells (P < 0.05, Dunnett’s test). [file peerj-03-1314-s003.png]

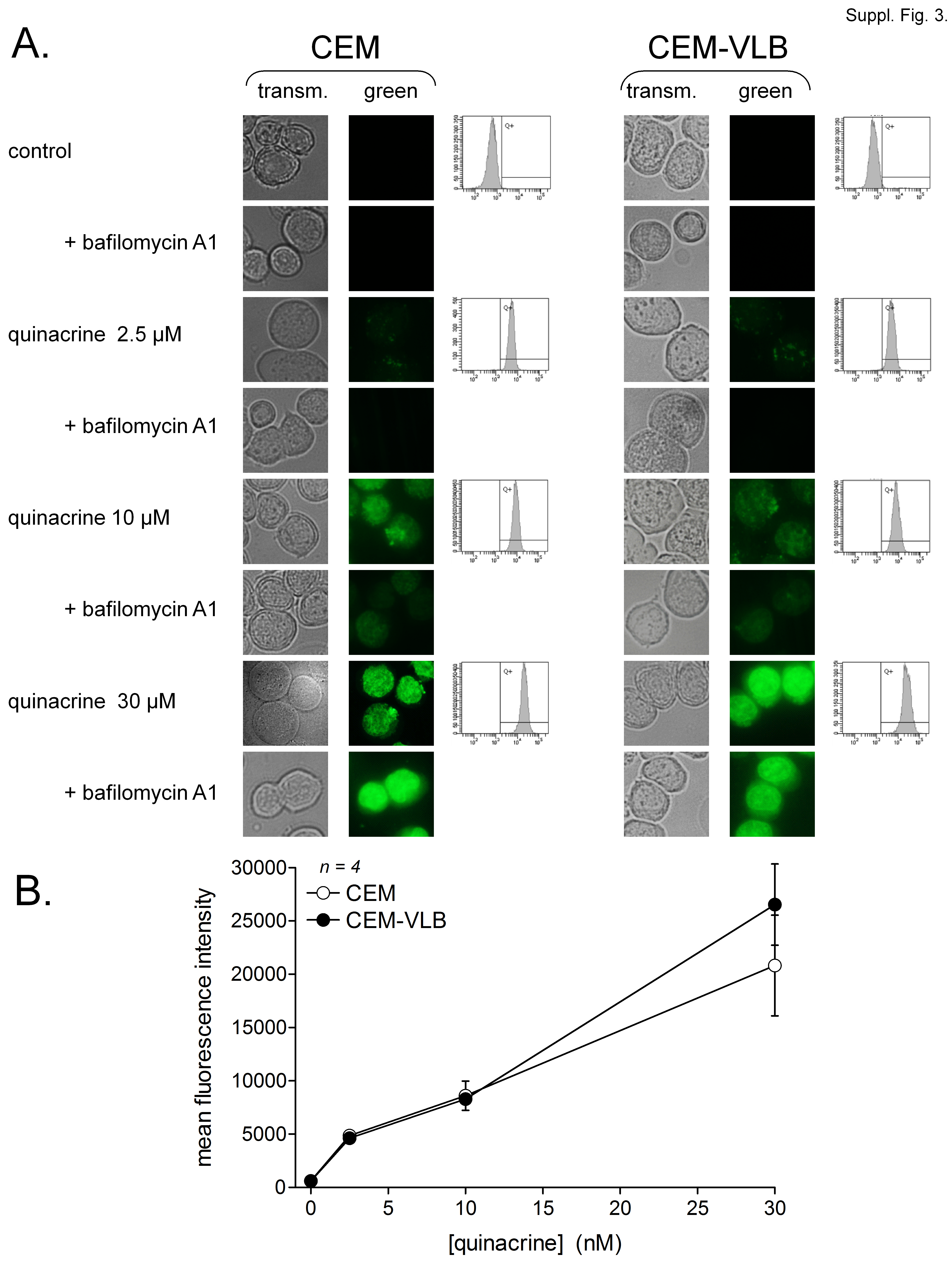

Supplement: Figure S3 — (A) Transmission and epifluorescence microscopy of representative cells (600×). (B) Cytofluorometric distribution of the drug-associated fluorescence in the 2 cell subtypes. [file peerj-03-1314-s004.png]

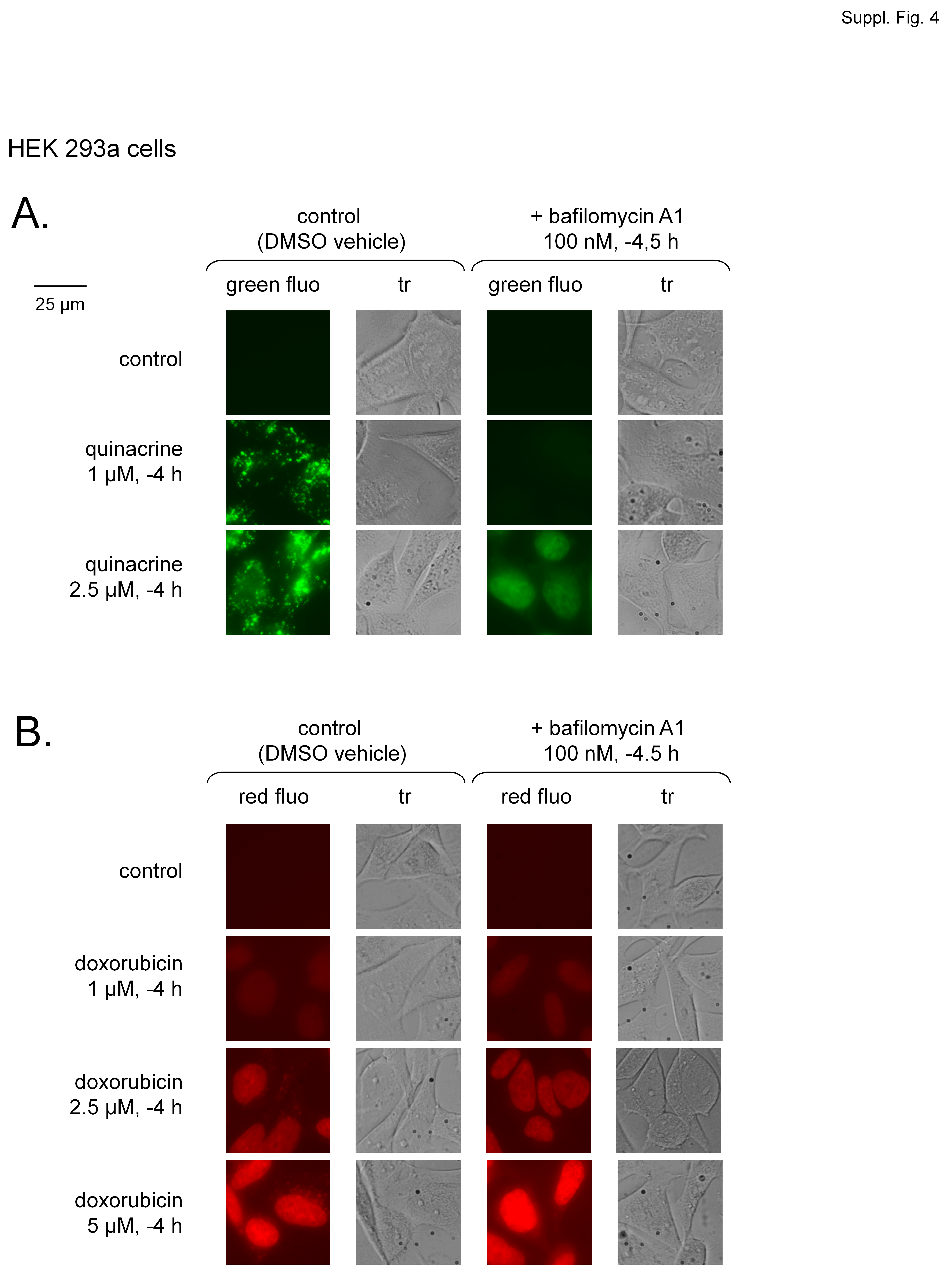

Supplement: Figure S4 — Morphological evidence of fluorescent drug uptake (A, quinacrine; B, doxorubicin; other treatments as indicated) in perinuclear vacuoles and/or nuclei of HEK 293a cells and effect of co-treatment with the V-ATPase inhibitor bafilomycin A1 on the drug uptake and subcellular distribution. [file peerj-03-1314-s005.png]

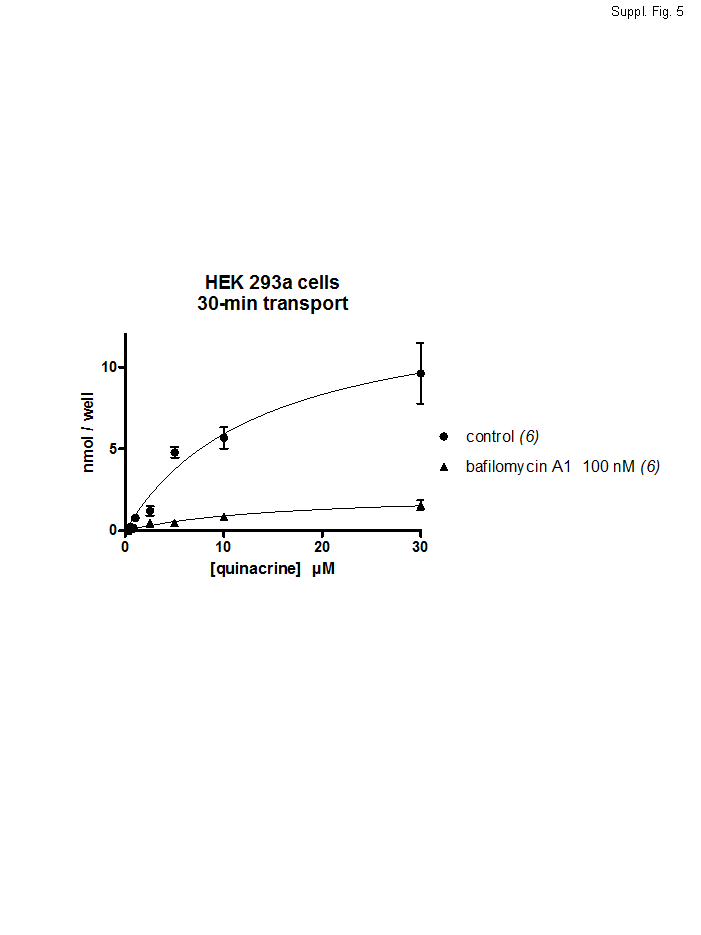

Supplement: Figure S5 — Uptake of quinacrine into HEK 293a cells as measured using extract-associated fluorescence: effect of quinacrine concentration on the uptake during a 30-min period as modified by an optional bafilomycin A1 co-treatment. [file peerj-03-1314-s006.png]

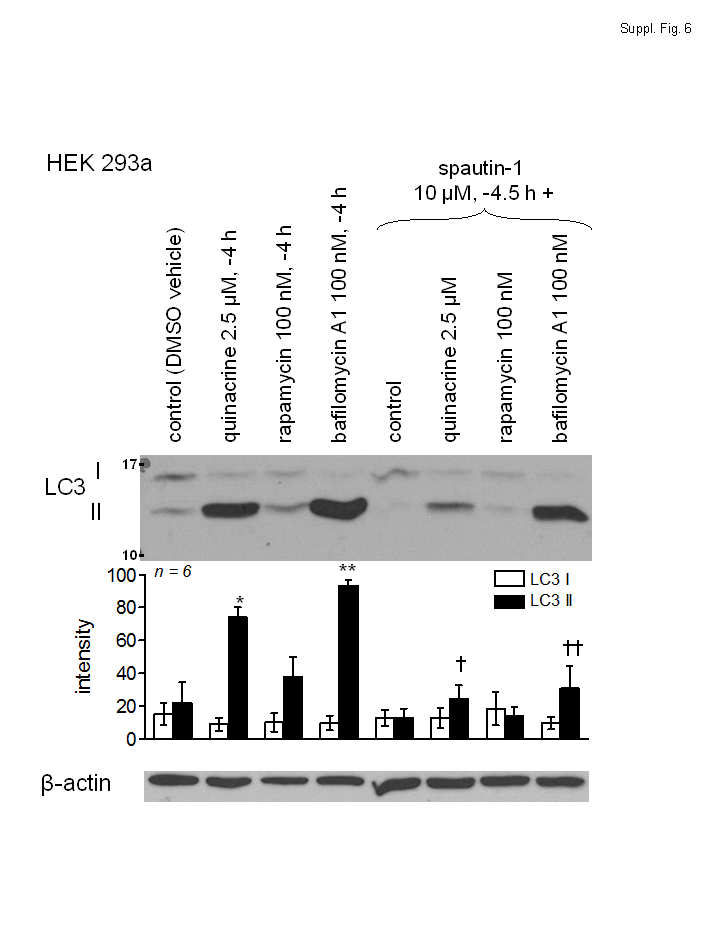

Supplement: Figure S6 — The figure includes a representative immunoblot of total cell extract revealed with anti-LC3 antibodies, a matched immunoblot for β-actin to document equal loading of tracks and the densitometric evaluation of LC3 I and II cell contents in replicated experiments and reported as histograms. The effect of the autophagic inhibitor spautin-1 was validated. LC3 I concentration between groups did not significantly differ (ANOVA). LC3 II values were heterogeneous (ANOVA P < 10−4; Bonferroni multiple comparison test for selected pairs: ∗ P < 0.01, ∗∗ P < 0.001 vs. control; † P < 0.01, †† P < 0.001 vs. same treatment without spautin-1). [file peerj-03-1314-s007.png]

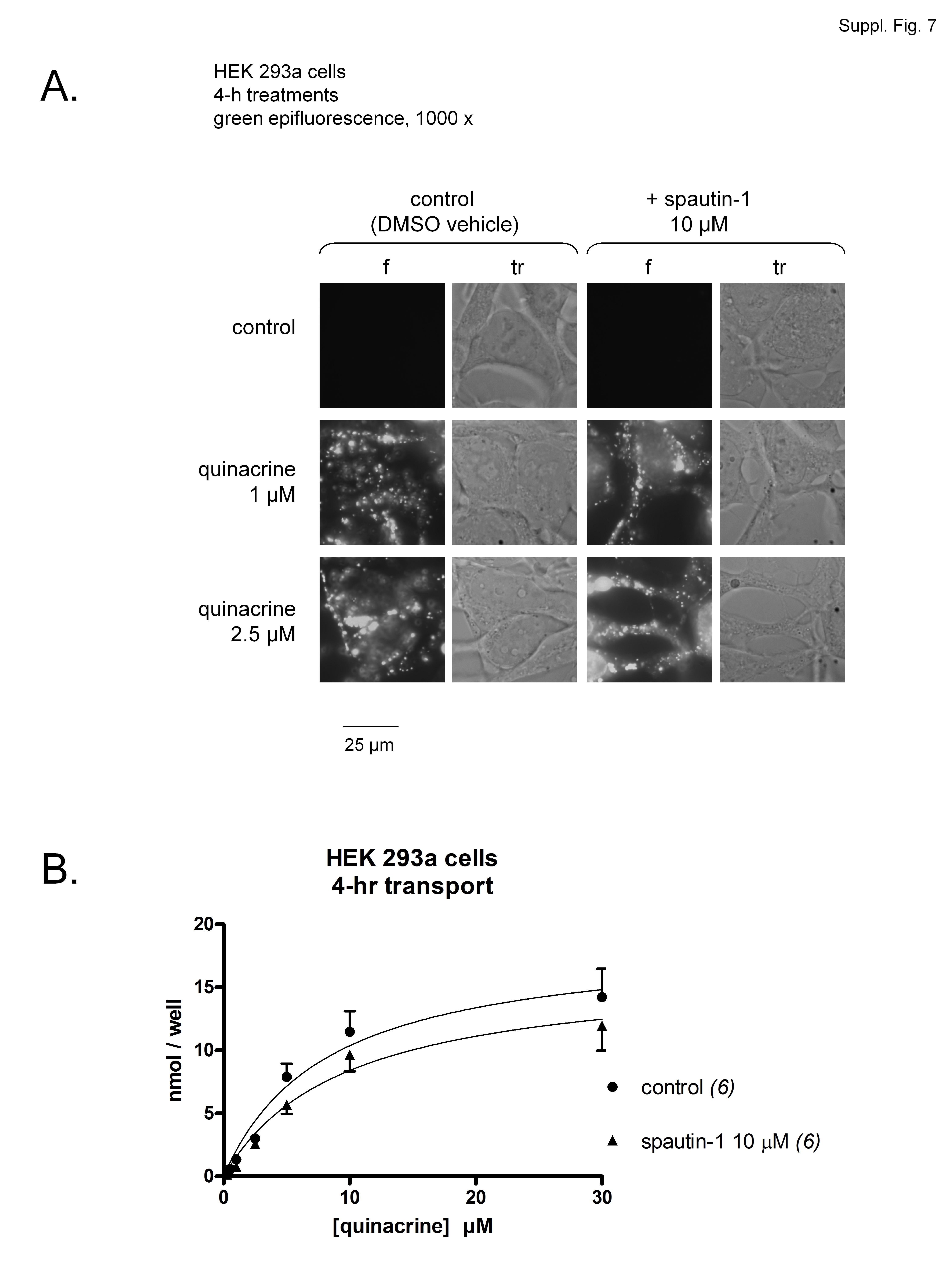

Supplement: Figure S7 — (A) Microscopy. Presentation as in Fig. S4. (B) Transport evaluated using fluorescence of cell extracts (presentation as in Fig. S5). [file peerj-03-1314-s008.png]

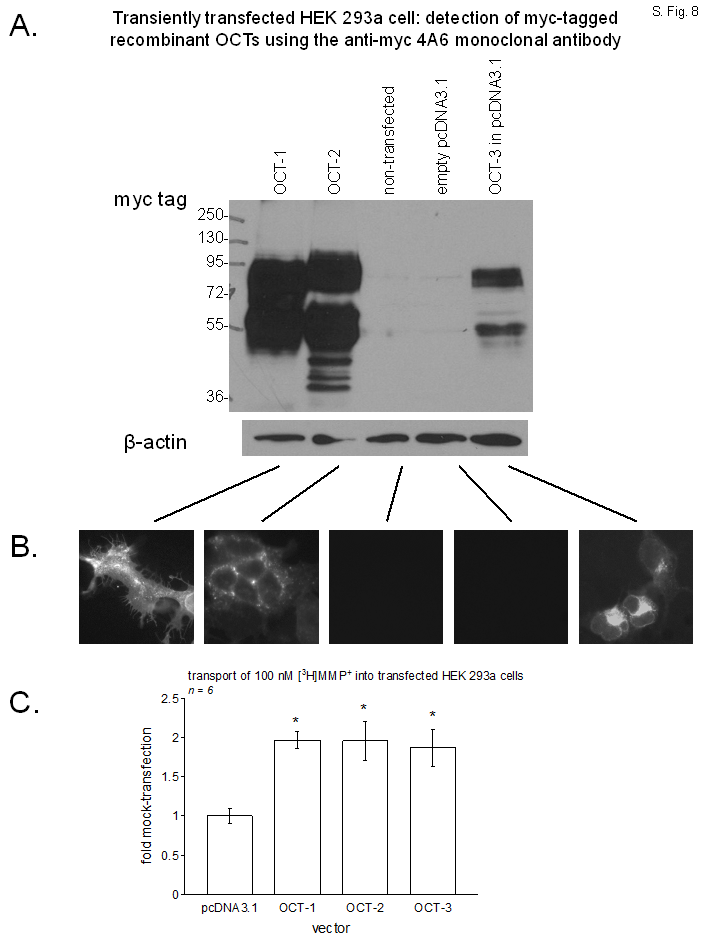

Supplement: Figure S8 — (A) Immunoblot for the myc tag of the constructions. Representative of 2 experiments. (B) Direct immunofluorescence (AlexaFluor-488 conjugated anti-myc tag antibody) of fixed and permeabilized HEK 293a cells transiently expressing OCTs. (C) Uptake of MPP+ (100 nM) as influenced by the expression of OCTs. Results are expressed as fold of the values recorded in mock-transfected cells (absolute average value 283 ± 53 fmol/well). Values were significantly heterogeneous (ANOVA, P < 0.01). ∗ P < 0.01 vs. mock-transfected cells. [file peerj-03-1314-s009.png]

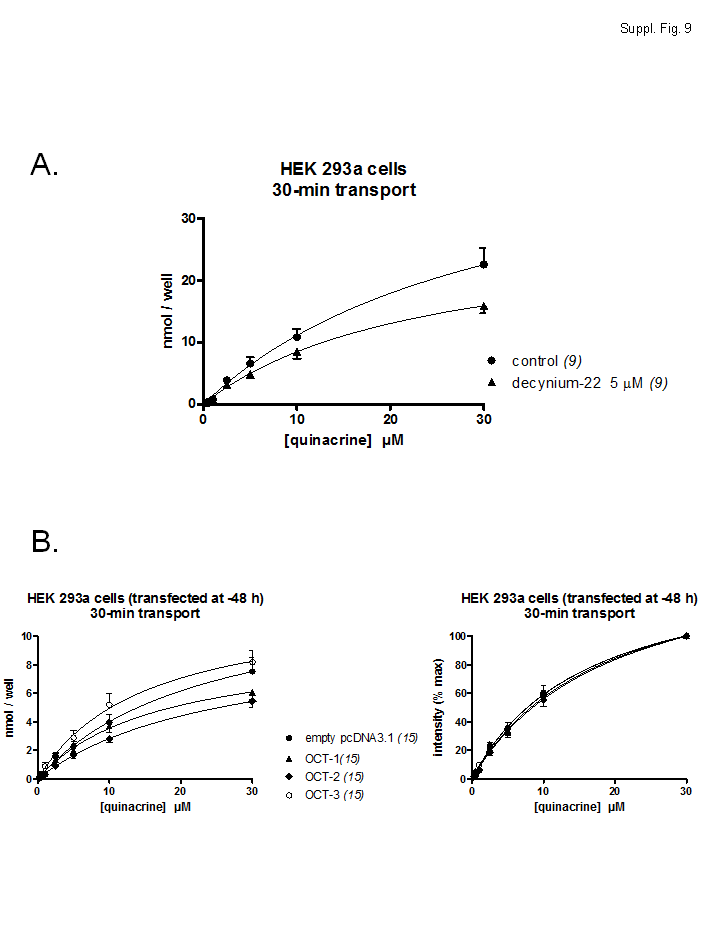

Supplement: Figure S9 — (A) Effect of the multivalent OCT inhibitor decynium-22 on the transport of quinacrine in total HEK 293a cell extracts. (B) Effect of overexpression of myc-tagged OCTs on the transport of quinacrine by transfected HEK 293a cells (expressed in absolute values, or as a percent of the maximal transport recorded at 30 µM; no significant differences were observed). [file peerj-03-1314-s010.png]

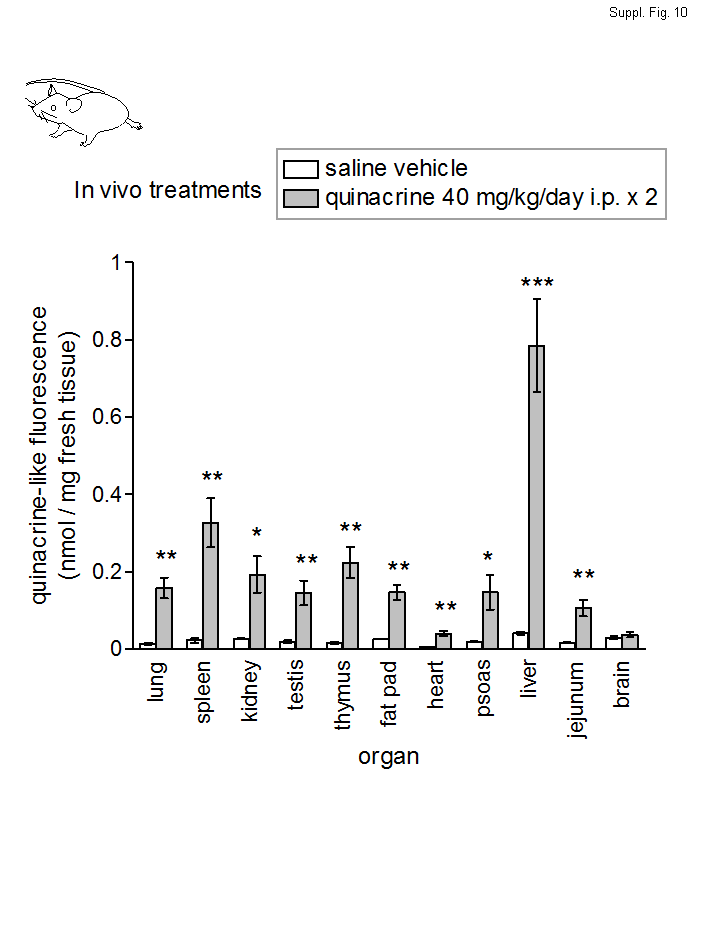

Supplement: Figure S10 — The autofluorescence is quantified in the extracts of saline-treated animals and was compared to the fluorescence in organ extracts from drug-treated animals using Student’s t test (∗ P < 0.05; ∗∗ P < 0.01; ∗∗∗ P < 0.001). [file peerj-03-1314-s011.png]

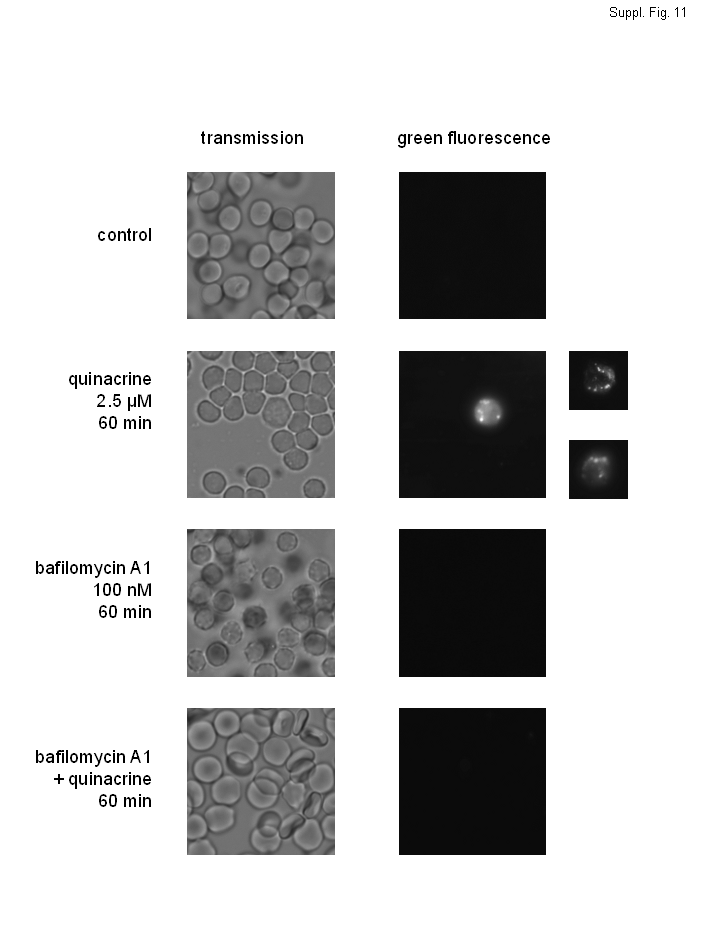

Supplement: Figure S11 — Quinacrine uptake, judged from fluorescence, is not present in red blood cells, but present in scattered leukocytes. Original magnification 600× (inserts at the right show some of the observed leukocyte morphologies). [file peerj-03-1314-s012.png]
